# Emotion regulation in patients with somatic symptom and related disorders: A systematic review

**DOI:** 10.1371/journal.pone.0217277

**Published:** 2019-06-07

**Authors:** Zeynep Emine Okur Güney, Heribert Sattel, Michael Witthöft, Peter Henningsen

**Affiliations:** 1 Klinikum rechts der Isar, Department of Psychosomatic Medicine and Psychotherapy Technical University of Munich, Munich, Germany; 2 Johannes Gutenberg University of Mainz, Department of Clinical Psychology, Psychotherapy and Experimental Psychology, Mainz, Germany; Italian National Research Council, ITALY

## Abstract

**Background:**

Somatic symptoms and related disorders (SSD) are prevalent phenomena in the health-care system. Disturbances in emotion regulation (ER) are commonly observed in patients suffering from SSD.

**Objectives:**

This review aimed to examine ER processes that characterize SSD by a systematic analysis of the available empirical studies.

**Data sources:**

PsycINFO and PubMed databases for the articles published between January 1985 and June 2018.

**Search terms:**

“emotion/al regulation” or “affect regulation” and various forms of SSD.

**Study eligibility criteria:**

Empirical studies that a) assigned adolescent or adult patients suffering from SSD based on a clinical diagnosis, and b) examined the relationship between ER and SSD, were included.

**Study synthesis methods:**

A tabular summary of the articles was generated according to study characteristics, study quality, variables, and findings. The findings were organized based on ER variables used in the articles and diagnoses of SSD, which were then re-organized under the main constituents of ER (attention, body, and knowledge).

**Results:**

The findings of the 64 articles largely supported the association between SSD and disturbances in ER, which are usually shared by different diagnoses of SSD. The results indicate that patients show a reduced engagement with cognitive content of emotions. On the other hand, bodily constituents of ER seem to depict an over-reactive pattern. Similarly, the patients tend to encounter difficulties in flexibly disengaging their (spontaneous) attention from emotional material.

**Limitations:**

There is a scarcity of longitudinal designs, randomized controlled trials, experiments, and diary studies suited to investigate the short- and long-term causal relationship between ER and SSD. Symptoms of SSD and measures to assess emotion regulation are heterogeneous.

**Conclusions and implications:**

Assessment of ER processes is potentially useful to understand SSD and for treatment planning. Furthermore, a concurrent investigation of the dynamic interaction of the ER modalities promises insights for better understanding of the role of ER in development, course, and maintenance of SSD.

## Introduction

### Somatic symptom and related disorders

Somatic symptom and related disorders are characterized by one or more bodily symptoms that are accompanied by excessive thoughts, feelings and behaviors [1]. The most common symptoms include pain in different parts of the body (back, joint, head, chest, etc.), disturbances in the organ functions (gastrointestinal, respiratory, etc.), fatigue, and exhaustion [2–4]. These symptoms cannot be adequately attributed to conventional organic diseases, structural changes in the body, or biochemical abnormalities [2,4–6]. The patients usually suffer from multiple physical symptoms, as well as comorbid mental and psychosocial disturbances, which precipitate and maintain the symptoms [2,7,8]. In case of a chronic course, the symptoms become difficult to treat and cause impairments in patients’ lives, resulting in high direct and indirect costs (increased health-care demands, working disability, and early retirement) [9].

As a consequence of the complex clinical presentation of SSD, the patients are diagnosed and treated by different health-care specialties, depending on their overriding complaints and the referral source. In general medicine, the symptoms are captured as “functional somatic syndromes,” such as “irritable bowel syndrome” for abdominal pain and altered bowel problems, “fibromyalgia” for musculoskeletal pain, joint pain, and fatigue (see Brown, (2007) [10], for the diagnostic labels for common symptoms by specialties). In conventional mental health, the symptoms are classified as somatoform disorders or recently, “somatic symptom disorders” [1,11]. *“*Medically unexplained symptoms” is another widely used term, although its use is contentious, as it is inadequate to represent the biopsychosocial nature of the complaints, it splits mind and body, and it constitutes premature labeling [2,10,12].

The classification of SSD is quite complex because of the extensive commonality between different syndromes of SSD. The diagnostic criteria for different syndromes typically overlap: Patients with a specific diagnosis usually also meet the criteria for another [8,13]. Also, different diagnostic groups share certain non-symptom characteristics, such that a majority of the patients are women, suffer from comorbid emotional disorders such as anxiety or depression, and report a childhood history of trauma [7,8]. The etiology, pathology, and neurophysiology of different diagnoses also show similarities [13]. What is also notable is that patients benefit from similar forms of treatment without regard to the specific symptom profile [14–16]. Such commonalities impede a clear-cut diagnosis and are complicated by the fact that criteria are not well established to differentiate symptom presentations and diagnoses [2,8,10,17].

Related to the difficulties in classification of SSD, there has been a debate between so-called “splitting” and *“*lumping” views. The splitting perspective holds that separate diagnoses for different symptom pictures should be given, because commonalities between diagnoses do not describe all patient groups, and lumping the disorders would not adequately explain the diversity and heterogeneity in patients’ symptom presentations. The lumping perspective, on the other hand, draws attention to the mentioned similarities among separate diagnoses and claims that commonalities are greater than differences between them. “Lumpers” note that a single syndrome receives different labels depending on the medical specialty and propose lumping the syndromes along a continuum [2,5,8,13,18]. Their concern is that clinicians and researchers focusing on one specific diagnostic entity tend to classify the symptoms as isolated from other biopsychosocial factors. Recent evidence seems to support both views, by showing both commonality and heterogeneity within and between diagnostic groups [17].

In order to avoid the terminology complications mentioned above and ensure comprehensive and integrative inclusion criteria, in this paper we examined the whole range of somatic symptom and related disorders that includes different functional somatic syndromes and medically unexplained symptoms (SSD) [5,19]

### Emotion regulation in patients with somatic symptom and related disorders

Theories about SSD acknowledge disturbances in emotion regulation (ER) processes as one of the psychological aspects contributing to the development, progression, and treatment of the symptoms [7,20–23] (see [24] for a review of the theoretical models). Empirical and clinical reports also quite consistently confirm the association between disturbances in ER processes and SSD (e.g., [25–27]). However, the underlying mechanism of this association is not clearly established. An overview of the theoretical accounts of SSD suggests that ER in SSD is characterized by an incoherent functioning of emotional response systems (cognition and body) during the regulation of emotions [20–24,28,29].

Biopsychosocial accounts of SSD include a developmental course of disturbed early interpersonal interactions, insecure attachment styles or trauma history as risk factors for the development of emotion regulation disturbances in patients with SSD. This socio-emotional trajectory has been found to be associated with alterations in the endocrine, immune, and pain-regulating systems [2,23,24,30]. Accordingly, psychotherapies for SSD incorporate interpersonal aspects and emotion regulation training into the therapy planning (e.g., [15,31–33]).

A relationship between ER processes and SSD is also supported by neurobiological studies highlighting the substantial neuronal connectivity and overlap of the emotional, somatosensory, and motoric subsystems [34–38]. These networks communicate closely with the autonomic and immune system and hypothalamic–pituitary–adrenal (HPA) axis, which also play key roles in awareness of internal body sensations and homeostatic regulation in response to emotional changes [36,39,40].

### Constituents of emotion regulation

How emotional processes are conceptualized certainly guides the pathway of ER research. Contemporary approaches to emotional processes draw attention to its systemic and dynamic nature, which consists of components interacting with each other at multiple levels, mainly physiological, cognitive, and behavioral (expressive). This perspective highlights the importance of research focusing specifically on these components and how they interact during ER [41–45].

According to this view, an emotional process is an inherently regulatory system that consists of its components operating through dynamic systems principles, such as continuous feedback mechanisms and circular causality (i.e. multi-directional and recursive relationship between elements of a system) [41,45–47]. This view postulate that, in response to an emotional perturbation, the components of emotion, such as appraisals, action tendencies or arousal, are continuously updated and regulated based on internal (e.g. proprioceptive) and external (e.g. cultural norms, socialization) feedback sources [45,48]. As a function of the continuity in feedback mechanisms, in the emotion process, the temporal distinction between emotion generation and regulation cannot be clearly distinguished [46,49]). Hence, emotion regulation goes beyond deliberate and effortful regulation strategies that are assumed to take place after the emotion is generated [50,51]. Instead, emotion regulation can be held as a suite of effortless and effortful processes, with or without a conscious supervision that changes the “spontaneous flow of emotions” [52]. When ER is not adaptively and flexibly employed, disturbances occur in emotional functioning [53]. As previously stated, no ER “strategy” in its broad sense, is healthy or unhealthy per se, without considering its context [53]. However, a dimensional approach that examines over- and rigid use of certain ER “strategies”/processes, such as suppression, disconnection from emotional experiences, or hypo- or hyper-reactivity in the components of ER [54] or even reappraisal [53] can define disturbances in ER.

In order to better understand ER, as previously remarked, “…we first need to know what is being regulated” [55]. During the regulation of emotions, what are indeed being regulated are the constituents of emotion. For example, expressive suppression of anger primarily requires the regulation of bodily behavior [56]. Or rumination basically involves a perseveration of attention on negative thoughts and feelings [55]. Catastrophizing mainly comprises a dysregulation of appraisals and thus overreliance on negative thoughts and feelings; techniques for breathing and muscle relaxation primarily regulate the body [52]. Certainly, ER being a dynamic process, multiple constituents of emotional process are simultaneously engaged; however, particular components are predominantly active depending on the form of ER process.

Given that ER occurs through the regulation of its constituents, identifying how each of them operates and interacts in an emotional context would help us to elucidate the ER process more precisely. Such an approach to ER, which looks closer at its constituents or targets, was implemented in previous studies and demonstrated its empirical and clinical utility [27,44,52,57,58]. For example, Koole (2009) [52], classified ER processes in terms of targets/constituents of ER (e.g. attention, body and knowledge), as well as its functions. He has gathered ER constituents such as action tendency, arousal, reflection, evaluation, beliefs, etc. around 3 overarching categories, “attention, body and knowledge”, thus refined the complex components into a practicable taxonomy. This approach offers high heuristic and experimental potentials to understand the mechanisms of ER. Moreover, in the context of SSD, classifying ER based on its targets is compatible with theoretical accounts and empirical evidence, which indicate a disintegrated interaction between cognitive and bodily components of ER in SSD [20,21,23,24]. In fact, with the recent progress in neuropsychobiology, it has been highlighted that concordant functioning of ER modalities is vital for healthy bodily functioning [36,37,59,60].

### The present study

The growing interest in ER and the body-mind connection in the past decades has yielded an accelerating number of studies that examined the association between ER and SSD. As these empirical findings accumulate, the need has grown for a systematic investigation of the literature that can explain and specify the relationship between ER processes and SSD. Our research was mainly guided by the central question: “How do patients with SSD regulate their emotions?” In order to pursue this goal, we specifically posed the following descriptive (Question 1), exploratory (Questions 2 and 3) and inductive (Question 4) questions: 1) Which ER processes were examined in different diagnoses of SSD? 2) Did the articles show a relationship between ER processes, and somatic and psychological symptoms in SSD? 3) Do these ER processes show differences between patients with SSD, healthy controls and patients with other mental or physical disorders? 4) What are characteristic ER processes in patients with SSD? In other words, how does ER operate in attentional, bodily, and knowledge domains in SSD? The fourth question is aimed to clarify the foremost question of the present review and will be elaborated in the discussion through the inferences drawn from the existing study findings.

The primary object of interest of this review is emotion regulation processes. When conceptually needed, we use the term “disturbances in ER” or similar terms. “ER constructs” denominate specific latent domains examined in the included studies (such as emotional awareness, expressive suppression, etc.) and “ER variables” the observed variables of each study.

## Method

### Literature search and inclusion of the articles

We conducted this review following the PRISMA guidelines for systematic reviews [61] (See “S1 Checklist”). We carried out a systematic search in PsycINFO and PubMed for articles published between January 1985 and June 2018. Due to the paucity of empirical research on emotion regulation before mid-eighties [62] we chose 1985 as a starting point for our search. We did not pre-register or make a formal protocol of the review publicly available.

The search terms included the keywords “emotion regulation” or “emotional regulation” or “affect regulation” and various forms of SSD. The keywords for SSD were established based on a previous meta-analysis [63] and a recently updated national guideline for SSD [5] (see “S1 Appendix” for a list of the search terms for SSD). Additional records identified through other sources and studies that examined emotion regulation despite their primary focus on alexithymia were also included.

The screening and selection of the studies was conducted by two authors of this review (ZEOG and HS). In the initial screening of the articles, the two authors checked the titles and abstracts and eliminated papers that clearly did not satisfy the inclusion criteria. The same authors then independently read the full text papers to determine the eligibility. Any disagreements or uncertainty were settled by a discussion and were resolved with a third author (PH) if necessary. Studies that assigned adolescent or adult patients (minimum age of 13) suffering from SSD based on a clinical diagnosis were selected for the review. We included a study if it either: (a) reported a relationship between ER and SSD, or (b) compared ER processes in patients with SSD with those of patients with other mental or medical disorders, or healthy subjects. All study designs (cross-sectional, case control, longitudinal, and experimental) except case studies were included. Dissertations and non-empirical papers, such as reviews, commentaries, expert opinions, and book chapters, and non-English papers were excluded. Brain-imaging and psychotherapy studies, which are beyond the scope of our review, were not included. As previous reviews on the relationship between alexithymia and somatization already exist, we excluded studies having examined only alexithymia as an ER variable (e.g., [26,28])

Following the initial screening, full-text articles were further examined for their eligibility. At this stage, relevance, sample details and measures of the studies were re-examined in the full text. Studies that assigned only a healthy sample or patients without an SSD diagnosis were excluded. In addition, the measurement methods, experimental procedures, and single items of the scales used were examined, in order to check their eligibility related to content validity (e.g., whether the instructions or the content of the experiment or scale items really measure ER vs. a more general regulation-construct, such as coping). If the scales were not provided in the article, they were examined in the original source of the scale. Articles presenting only a global score of an emotion regulation-related construct that did not allow identification of specific ER Subscale scores were excluded (e.g., those providing only the findings on coping style, but not on the reappraisal subscale). If a study examined merely regulation of somatoform symptoms without any emotional reference, it was excluded (e.g., pain regulation, avoidance of painful activities, catastrophizing the symptoms, or rumination about the symptoms). Studies that assessed only feeling tone/affective experience but not ER were excluded. Finally studies that did not report a relationship between ER variables and SSD were excluded.

### Organization of the articles

The articles were organized based on (a) ER variables referred to in the article, and (b) diagnoses of SSD. They were then relocated under the overarching categories of attention, body, and knowledge [52], through investigation of the measures and the findings. When locating the ER processes, we implemented a combination of bottom-up (empirical) and top-down (theoretical) methods, which renders it a robust classification approach [58]. In other words, besides the guidance of previous studies [52,57] and models [41,45,50] in defining the constituents and classification of the ER processes (top-down), the examined ER variables and their measurement method in each study contributed to the classification (bottom-up). We examined the operational definition of each ER term assessed in the article, the corresponding measurement method, and the experiment instructions/procedures or single items of the administered psychometric scales implemented for the assessment of ER. The original sources of the measures were also consulted and listed in a separate reference section (see “S1 File”). The first and the second author built the organization system. In cases of indecision about locating the ER variables, they discussed the cases and settled on a consensus. The outcome of this organization, including emotion regulation variables and their assessment methods, are presented for the attention, body, and knowledge domains in “S1 Table”.

#### Attention

Attentional regulation is phylogenetically and ontogenetically one of the earliest mechanisms for dealing with affects [38,60]. Infants’ gaze aversion, parents’ visual orienting or rocking of infants to reduce distress and alert behavior, are examples of early forms of attentional regulation of affect [38,64]. In response to affective perturbations, attentional, affective, and autonomic systems operate in a highly interconnected functional and structural network, whereby the organism allocates its resources for organization and selection of responses, and for psychophysiological modulation [64–67]. Attentional regulation of emotions can be automatic or deliberate, which involves alerting, directing one’s attention towards or away from the emotional material, or modifying the attention process [52,56,64,68]. Previous studies suggested the following attention-oriented emotion regulation processes: attention deployment, flexibility or hypervigilance in attention, thought suppression, attentional avoidance, rumination, distraction, focusing on the positive, meditation, and mindfulness [52,57,58].

#### Body

The vital role of bodily processes in the experience and regulation of emotions has been consistently confirmed. Research across different disciplines continues to converge on the bidirectional role of afferent and efferent feedback in the experience and regulation of emotions [39,69,70]. Bodily constituents of emotion involve indicators of arousal, action tendency, and embodied means of emotional behavior such as facial and bodily expressions of emotion [41,45]. The following regulation processes are considered in this domain: sympathetic and parasympathetic autonomic activity; startle responses; involuntary muscle movements; vocal, facial, and postural behavior; bodily relaxation; stress-induced eating [45,52,71].

#### Knowledge

Knowledge-oriented emotion regulation is the most widely studied form of emotion regulation. It involves conscious or unconscious appraisals and attribution of emotionally significant events, such as the relevance, implications, significance, and meaning of the event [45,72,73]. Emotion regulation styles, such as catastrophizing, reappraisal, acceptance, emotional awareness, emotional clarity, and ability to reflect and distinguish emotions are considered in this domain [52,57,58]

### Data analysis

Because of the large heterogeneity of the studied patient groups, ER variables, and methods, no statistical comparison by a meta-analysis was possible. It is expected that our qualitative findings can pave the way for future studies with specific research questions suitable for a quantitative review.

For the narrative analysis, a preliminary synthesis was first developed using tabulation of the data according to the study characteristics, study quality, variables, and findings. Emotion regulation variables and their measurement methods were specified at this stage. The findings were organized based on ER variables and diagnoses of SSD (for research questions 1 to 3). At the second stage, the articles were relocated under the categories of attention, body, and knowledge for interpretation of the findings (main goal of the study, for the research question 4). Finally, the findings of the articles were summarized and discussed in a theoretical framework.

### Quality appraisal

The quality appraisal followed a systematic procedure, which is a modified version of the one conducted by Brown and Reuber [74]. Because an established quality rating system in this field did not exist at that time, the authors developed and validated a modified catalogue, which we modified slightly in order to cover the specific needs of the review presented here. We followed a procedure similar to that of Brown and Reuber [74] for assessing the criteria related to: (1) design, (2) sample size adequacy (based on considerations of effect size and the power criteria of Cohen [75]— i.e., how large an effect size had to be detected: very small < 15 participants, small < 26 participants, moderate 26–63 participants, and large ≥ 64 participants, in each group), (3) consecutive sampling (yes/no or not stated clearly), (4) use of standardized measures (yes/no), (5) type of comparison groups, if available, and (6) demographic matching of the comparison group (yes/no or post-hoc analysis). [75]

We added four other criteria that we considered relevant to assess the included articles, which had assigned heterogeneous diagnoses. The quality appraisal was amended accordingly by the following criteria: (7) specificity of the diagnostic labels (specific, e.g., psychogenic non-epileptic seizures vs. broad, e.g., medically unexplained symptoms), (8) the presence of sufficiently reported inclusion and exclusion criteria (yes/no), (9) the use of established diagnostic criteria (yes/no/not available), and (10) the application of more powerful inferential statistics, as opposed to more explorative and descriptive statistics (yes/no). Well-reported in-/exclusion criteria and the use of established diagnostic criteria should make it possible to recruit other samples from the population in question with a high probability of substantial concordance, and to generalize conclusions from the respective study to the larger populations for those patients. Finally, we introduced a distinction between the use of explorative statistics (when only means, standard deviations, or simple correlations were provided) vs. inferential statistics (controlling for potential confounders, application of higher order/interaction analyses). While the former allow-in a strict sense- conclusions related to the observed sample only, the latter provide conclusions and tests of hypotheses related to properties of a population, by assuming that the observed data stem from a larger population [76].

As the original rating system developed by Brown and Reuber [74] had already demonstrated high interrater reliability, we determined the reliability of the added criteria applying a similar approach. 12 articles were independently rated by ZEOG and HS resulting in a substantial overall interrater-reliability for those three items (Cohens Kappa κ = 0.78). The remaining empirical papers were then evaluated by HS, and any uncertain ratings of the remaining articles were discussed and agreed with ZEOG subsequently.

We finally calculated a total quality score defined as the proportion of criteria items rated as “yes” (items 3, 4, 6, 8, 9, 10). This score refers to a total of 4 to 6 items (depending on whether established diagnostic criteria exist and/or a comparison group was examined, which could be matched demographically). Quality of the studies was rated with +, ++, or +++ when 25–49%, 50–79%, or 80% or more of the criteria were rated with “yes.”, following the suggestion of Brown & Reuber [74]

## Results

**Fig 1:**
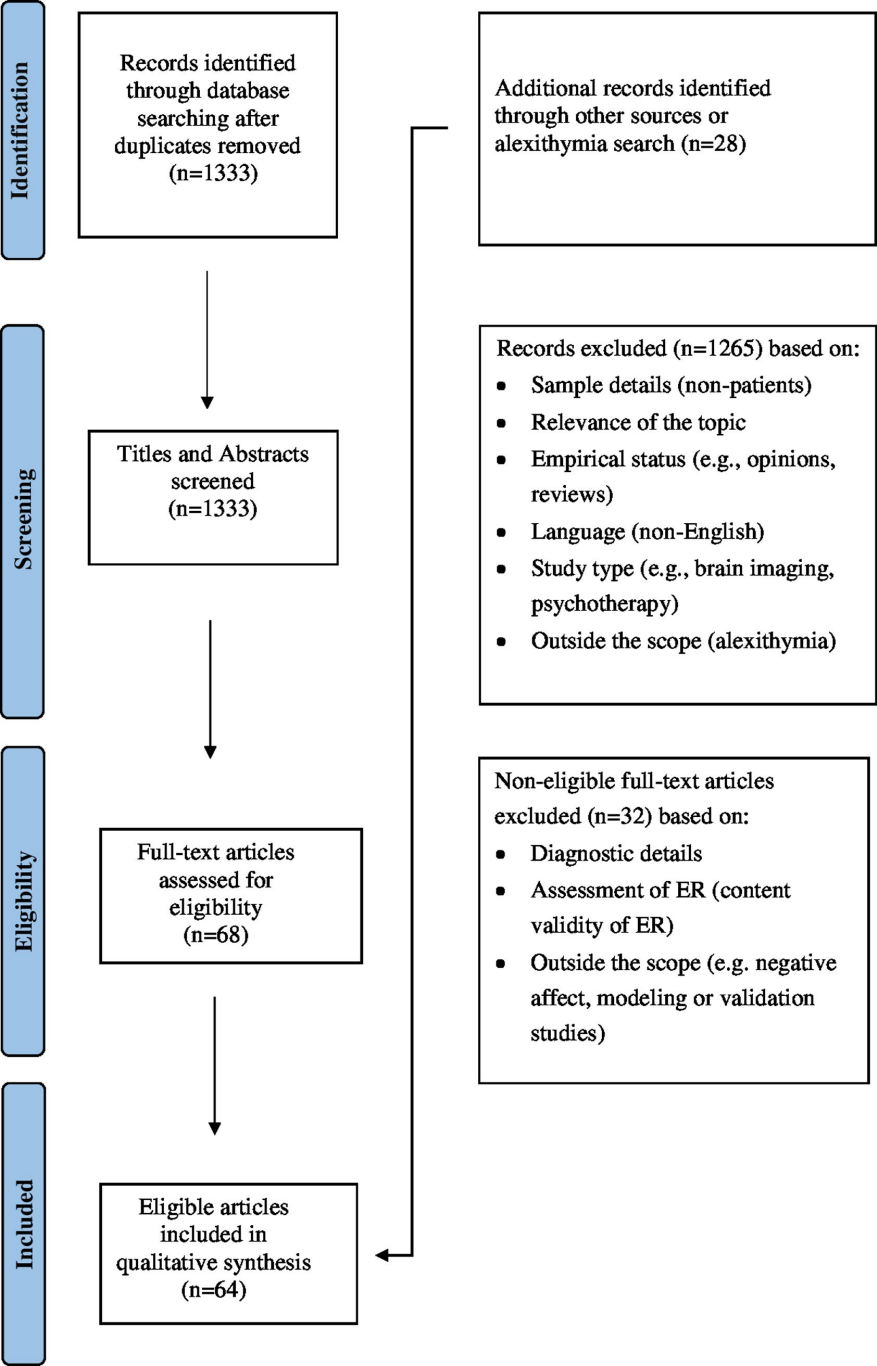
Information flow on study selection.

The flow diagram for the 64 included articles is shown in Fig 1.

### Quality appraisal

The characteristics of the included studies displayed a large variability. The majority of the studies (42; 68.9%) included patients with specific diagnostic labels, e.g. functional syndromes, and most of those studies (32 out of 43; 74.4%) used established diagnostic criteria for those. For broadly defined diagnostic labels well defined criteria were not available or not applied more often (10 out of 19; 52.6%). Nearly two-thirds of the studies reported inclusion criteria sufficiently to allow a replication (38; 62.3%). More than a half of the investigations reported multilevel outcomes, whilst the remainder provided patient reported outcomes only, using consistently validated instruments (with the exception of a qualitative study applying grounded-theory methodology). 46 studies compared the diagnostic label under question with one or more other samples, most often with healthy controls (43; 70.5%), and in a few cases with other medical conditions (7; 11.5%) or both. 20 out of those 46 studies (43.5%) matched the examined samples according to sociodemographic data. 15 studies (24.6%) reported a consecutive recruitment mode. For statistical analyses inferential approaches were used in 32 (52.5%) of the studies. Sample sizes varied from very small to large and were in 41 studies at least moderate (67.2%). Small sample sizes were more common in studies applying experimental approaches. The total quality score, defined as the proportion of preferable criteria items rated as “yes”, indicated that the majority of studies fulfilled 50% or more of those criteria (50, 82.0%). Two studies did reach less than 25%, indicating a low methodological or empirical quality of the study. (see “S2 Table” for the table of quality appraisal)

### Diagnostic groups

The review covered various patient groups disturbed by: general somatic symptoms, chronic pain symptoms in the whole or some regions of the body and musculoskeletal system, fibromyalgia, fatigue, gastrointestinal, neurological, or motor/movement symptoms. Pain-related disorders have taken considerable attention compared to others; however, diagnoses were diverse, and their symptom presentations usually overlapped. Different ER variables were examined heterogeneously in various diagnostic groups and there was no systematic focus on a certain ER facet exclusive to a certain diagnosis. (See “S3 Table” for diagnostic groups and examined ER variables in each diagnostic group.)

### Emotion regulation variables examined in the articles

A wide range of ER variables were examined in patients with SSD: Attention switching, anger expression and suppression, autonomic nervous system activity, emotional expressiveness, emotional awareness, emotional theory of mind, emotion recognition, and emotional appraisals were among the most investigated ones. These variables were heterogeneously investigated in diverse diagnostic groups. (See “S4 Table” for examined ER variables in each diagnostic group.)

### Attention

Directedness and flexibility of attention to emotional stimuli, suppression of emotional thoughts, and facets of mindfulness (non-judgmental attention to and awareness of one’s current experience) [77] were among the researched topics of attention-oriented ER. Four articles have examined attention-focused ER with behavioral paradigms, whereas 11 articles examined it by self-report measures. (See “S5 Table”.)

#### Attention switching

Experimental paradigms for assessing patients’ attentional regulation of emotional stimuli pointed out patients’ attentional inflexibility and difficulty in disengaging from emotional material. In a study including patients with psychogenic non-epileptic seizures, patients took a longer time to switch their attention from the emotional dimension of pictures than from the age dimension, indicating more cognitive costs for emotional features of pictures. Healthy controls, on the other hand, showed equal switching performance for both age and emotion dimensions [78]. In patients diagnosed with psychosomatic disorders, it was likewise found that difficulty in switching attention from the emotional material was predicted by somatic symptoms, depression, childhood trauma, and dissociation [79]. These two studies also demonstrated a positive association between emotion suppression and attentional fixedness in both patient groups [78,79]. This relationship among emotion suppression, attentional fixedness, and somatic symptoms could be also supported by a study with chronic back-pain patients [80] and another one with chronic fatigue syndrome [81]. The first study reported that suppression of anger-related thoughts, which through an *ironic effect* deploys attention, influenced pain behaviors of the patients depending on patients’ trait anger regulation style [80]. In the second study, suppression condition also increased the level of anxiety in both patient and healthy groups but it did not affect self-fatigue of the participants [81].

#### Goal directedness when emotionally distressed

Based on a commonly used questionnaire for examining difficulties of emotion regulation (Difficulties of Emotion Regulation Scale, DERS) [82] patients with psychogenic non-epileptic seizures or conversion disorders reported greater difficulties in focusing on their tasks when they are distressed, compared to medical patients or a normative sample [25,83,84]. This difficulty also predicted gastrointestinal symptoms in patients with functional gastrointestinal disorders [85].

#### Attending to emotions

More effortful and conscious ways of attending to emotions were also focused by several studies. They found evidence of a negative relationship with certain aspects of SSD and consciously attending to emotions. For example, in patients with fibromyalgia, depression, anxiety, and neuroticism were negatively correlated with acting with awareness, such as doing things by paying attention [86]. Similarly, in patients with functional gastrointestinal symptoms, mindful attention negatively predicted depression, anxiety, and stress, and also negatively correlated with all facets of emotion regulation disturbances [85]. Mindful attention capacity was also shown to improve following psychotherapy, accompanied by improvement in SSD in patients with medically unexplained symptoms [87].

Nevertheless, two studies including patients with non-epileptic seizures and epilepsy, having used another questionnaire DERS [82] did not support these results. Patients’ evaluations of their capacity to attend to emotions and acknowledge them did not differ from those of patients with epilepsy or a normative sample [25,83]. The authors discussed a possible reliability or validity problem with this subscale [25]. However, another study, which used the same measure to compare patients suffering from conversion disorders with healthy adults, confirmed the capacity of attending to and acknowledging emotions to be reduced in the patient group with conversion [84].

When patients with medically unexplained symptoms (MUS) were compared with patients with MUS and comorbid major depressive disorders (MDD), with only MDD, or with healthy adults, the results seemed different. In this study, the only difference in the capacity of attending to emotions was reported between the MUS with comorbid MDD patients and healthy controls. The authors suggested that depression play a mediator role between difficulties in consciously attending to emotions and MUS.

### Body

Bodily-oriented ER processes were a relatively salient topic of the reviewed studies after knowledge-oriented ER processes. (See “S6 Table”.) The frequently researched themes were the overt facial and bodily expressions of emotions, especially anger expression; autonomic nervous system activity; affect-modulated startle in the muscles; and impulse control difficulties when emotionally distressed. While 26 articles examined body-focused ER by self-report measures, 16 articles implemented behavioral measures.

#### Anger expression and anger suppression

Patients’ anger expression style has drawn considerable attention. All of the anger expression studies except one [88] were conducted with pain and fibromyalgia patients, on the basis of possible shared neurophysiological pathways for anger expression and pain. The assessment of anger expression was based on self-report questionnaires, diaries, and experimental manipulations. All the studies except that with irritable bowel syndrome [88] found a positive association between excessive expressive anger suppression or uncontrolled anger expression and aspects of SSD. Habitual expression of anger in an uncontrollable manner (anger-out) was related to higher experimental acute pain intensity [89]. As an interesting biological observation, the study showed that beta-endorphin release, which was related to less pain being experienced, was negatively predicted by greater anger-out style in both patient and healthy groups. Daily anger expression, assessed by the diary method, was also elevated in patients relative to controls, which amplified their subsequent chronic pain intensity later in the day [90]. The diary method for examining anger expression was also employed for examining both patients’ and their spouses’ perceived criticism, hostility, and negative affect. This interpersonal study revealed that the gender of the patient is an important predictor of perceived criticism, hostility, and negative affect reported by both patient and spouse, such that for male patients, expressing anger elicits more negative reactions from the spouse [91]. Secondly, the study reported that, congruently with other studies, greater anger expression and inhibition are related to patient- and spouse-reported pain and pain interference of the patients [92].

In addition to higher trait anger-out, trait anger-suppression (anger-in) was greater in patients than healthy controls [93,94] and patients with “medically explained” pain [94]. Some positive association was also reported between anger-in and psychosomatic variables, such as end-of-day-pain [95], experimental acute pain intensity [89], state pain and pain interference [92], depression, alexithymia [93], and mental distress [96]. One interpersonal study found that greater anger inhibition was related to decreased concurrent spouse criticism, which indicates that patients might inhibit their anger expression in order to avoid criticism from the partner, at the expense of greater pain [91].

A mismatch between habitual anger expression style and state anger expression was shown to be decisive in predicting pain-related complaints in four studies. A diary study reported the lowest pain in the patients with high trait anger expression who actually expressed their anger [95]. Supporting this interaction, trait anger-out patients in the suppression condition displayed more pain behavior and symptom-specific muscle reactivity followed by the slowest recovery compared to their counterparts in the no-suppression condition [80,97]. Pain experience could also influence subsequent state anger expression, moderated by the trait anger expression style [90], suggesting that persons with high trait anger-out and anger-in styles are at risk for dysregulated anger expression when they are in pain.

#### Expressive suppression and emotional expression

Expressive suppression of emotions, measured by self-reports of patients, was also quite frequently reported in patients with SSD. Three studies of patients with psychogenic non-epileptic seizures or functional neurological symptoms examined self-reports of the patients regarding expressive control. These studies reported elevated control reactions especially for sadness and anxiety, in addition to expressive suppression in patients [78,98,99]. Another study with a broad diagnosis of SSD also reported a trend towards more expressive suppression in the patients compared to controls [100]. These findings were supported by a reduced self-report-based emotional expressiveness in the fibromyalgia patients [96].

Higher expressive suppression was associated with other psychological factors, such as poorer cognitive flexibility [78] negative affect, and mental distress [96]. It was also related to pain catastrophizing and mediated the relationship between negative affect and pain catastrophizing [101]. One study reported an interaction between the experience and suppression of emotions and somatic symptoms, such that patients who experience emotions intensely but suppress emotional expressions suffer most from the impact of symptoms [102]. Expressive suppression of emotions was shown to decrease following psychotherapy, which was accompanied by improvements in symptom reports [87].

In an interview-based study, patients with somatoform disorders reported a lower capacity to nonverbally express their feelings than controls, which was also positively correlated with dimensions of alexithymia [103]. Another qualitative study also reported patients’ tendency to bottle up emotions but not to express them, for reasons such as believing that expressing does not make a change, fear of rejection or not wanting to worry others [104].

Observable emotional behavior of patients with SSD was examined by three interpersonal paradigms. The characteristics of the non-verbal emotional expressions were shown to be quite negative according to two studies conducted with patients diagnosed with psychosomatic disorders, including somatoform disorders (however, the sample also included anxiety and depression patients) [105] and chronic pain disorders [106]. One of the studies reported that during therapist-patient interviews, patients displayed more facial contempt and negative emotions than controls, who displayed more genuine joy [106]. Moreover, these negative or aggressive expressions were associated with a low integrated personality structure [106] or lower alexithymic traits [105]. What is also notable is that, in response to patients’ negative displays, therapists’ facial expressions were also negative, especially that of contempt [105,106]. These findings illustrate that patients with SSD can facilitate certain types of negative interactional patterns in their social encounters. One study examined how the interaction partners’ validation and invalidation is related to patients’ pain complaints. It was found that patients’ gender interacts with behavioral expressions of validation and invalidation in predicting pain complaints. Only in couples with a male patient was reciprocal invalidation related to worse pain, but interestingly, not in couples with a female patient [107].

Computerized tasks, such as picture viewing or film watching, for observing emotional behavior were implemented in three studies with psychogenic non-epileptic seizures [108],irritable bowel syndrome [109] and chronic fatigue syndrome [81]. Roberts and colleagues [108] compared patients with psychogenic non-epileptic seizures, with seizure-free individuals with high or low posttraumatic stress symptoms, given the high rates of trauma history in the patients. The study reported a difference between the patients and the trauma-low control group only in positive emotional expressiveness, not negative ones, during positive, negative, and neutral picture viewing. Another study with irritable bowel syndrome reported that patients displayed more sadness and tended to display more rage than healthy controls while watching frightening films [109]. A third study with patients with chronic fatigue syndrome examined patients’ facial expressions also during watching stressful films for expression choice and suppression conditions. As opposed to the previous two studies, this study found lower number of expressed, as well as intensity of emotions in the patient group compared to controls, as rated by the observers. This effect remained significant after controlling for anxiety and depression [81].

In sum, although self-report studies point to a higher level of expressive suppression and limited expressivity, majority of the studies based on observed emotional behavior suggest that patients are not less expressive, except one study [81]. These studies reported that, patients with SSD tend to display more negative and fewer positive emotional expressions compared to healthy controls. Their interaction partner also tends to exhibit more negative emotions. Whether the diverse findings in observable emotional expression between studies stem from methodological or diagnostic differences should be further examined.

#### Autonomic nervous system activity

Research on the physiological parameters of emotion regulation has mostly shown aberrant autonomic functioning in patients with SSD. The findings indicate somatic vigilance and reduced capacity to down-regulate the autonomic reactivity to stress. In a recent study, even during a resting state, patients with chronic whiplash-associated disorders presented reduced heart rate variability (HRV). Remarkably, vagally mediated HRV was inversely correlated with pain catastrophizing, which was more elevated in the patient group than in the healthy controls [110]. Resting state aberrant parasympathetic functioning was highlighted in another study comparing psychogenic non-epileptic seizures with seizure-free individuals with low or high posttraumatic stress symptoms (PTS) [108]. Unexpectedly, patients did not differ from either group of seizure-free individuals when looking at emotional pictures. However, at baseline, patients displayed lower respiratory sinus arrhythmia than individuals with low PTS, but did not differ from those with high PTS. These two studies indicate a hypervigilant emotional system expecting a continuous threat and an inability to inhibit sympathetic activation.

Some other studies showed autonomic differences between baseline and emotion-inducing tasks. One study with somatoform disorders confirmed that during emotion-relevant tasks, patients displayed lower parasympathetic and increased sympathetic activation [71,111]. Similarly, patients with irritable bowel syndrome displayed a sympathetic withdrawal and increased heart rate from baseline to a fear-eliciting film session [109]. Such an increased sympathetic activation was also found in a study with patients with chronic fatigue syndrome. This study reported higher skin conductance during stressful film watching but not during baseline, which remained significant after controlling for anxiety and depression [81]. Moreover, increases in fatigue was positively related to skin conductance response during baseline and film watching only in the patient group Another study made a distinction between low vs. high levels of alexithymia and compared the autonomic functioning of the patients with persistent somatoform pain. This study reported lower skin conductance and greater negative affect in the high-alexithymic patient group. Nevertheless, the high- and low-alexithymic groups did not differ in terms of heart rate or muscle electrical activity across baseline, relaxation, or stress phases [112]. However, the study did not compare the findings with healthy controls.

Besides peripheral physiology parameters, affect-modulated startle in eye blink during emotional exposure was examined and studies showed congruent results with those that examined cardiac functions. Startle reflex is known to be mediated by the activities of the amygdala complex and is used as an indicator of emotional reactivity [113]. A study assessed eye blink reactivity in patients with psychogenic movement disorder and found higher and indiscriminate startle responses to both positive and negative pictures compared to neutral ones; however, controls displayed the highest startle responses to negative pictures followed by positive and neutral ones [114]. Another sample of patients with interstitial cystitis/painful bladder syndrome likewise showed a greater acoustic startle reflex than controls during non-imminent threat conditions (baseline, safe, and anticipation phases); however, patients and controls showed similar robust responses in imminent threat conditions [113]. This indiscriminate and rather vigilant bodily arousal in response to emotional stimuli was supported by another study of patients with irritable bowel syndrome, who reported greater pain for painful and non-painful rectal distensions in both stress and relaxation conditions, as compared to controls [115].

#### Impulse control difficulties

Impulse control difficulties indicate difficulties in controlling behavior when emotionally distressed. The studies that examined these difficulties (mostly by using self-report questionnaires) provide some insights about patients’ difficulty in down-regulating physiological markers of arousal and action tendency. Patients with psychogenic non-epileptic seizures reported greater impulse control difficulties compared to epilepsy patients [25] and normative data [83]. This difficulty was especially pronounced in the emotionally dysregulated patient group [25,83]. The difficulty of impulse control could also independently predict the gastrointestinal symptoms and anxiety of patients with functional gastrointestinal disorders [85]. Also in patients with conversion disorders, impulse control difficulties were more pronounced than in healthy controls and remained a significant predictor of patient status, after statistically controlling for other emotion dysregulation factors [84]. As opposed to marked impulse control difficulties reported in the literature, which represent a non-adaptive emotion regulation strategy, an action readiness to confront emotions was reduced in patients with medically unexplained symptoms, but only those with a comorbid major depressive disorder [116].

#### Emotional decision making based on bodily signals

One study examined fibromyalgia patients’ emotional decision-making ability in tasks requiring awareness of internal bodily signals associated with affective change (the Iowa Gambling Task). The study reported reduced performance in patients, characterized by more disadvantageous and random card selections than was the case for controls. However, patients’ performance on other standardized cognitive tests was comparable with that of controls, ruling out a cognitive difficulty responsible for patients’ reduced performance in the experiment [117]. When the capacity for perceiving the bodily sensations associated with emotions was examined, it was shown to be reduced compared to controls only in patients with medically unexplained symptoms (MUS) and a comorbid major depressive disorder (MDD), but not in those with only MDD or MUS [116].

### Knowledge

Twenty-five articles reported knowledge-oriented ER in terms of self-report based results, 15 articles reported behavioral results and two articles reported the both. (See “S7 Table”.)

#### Emotional awareness and emotional theory of mind

Patients’ awareness and capacity to understand their own and others’ feelings was the most-studied subject of knowledge-oriented emotion regulation. These capacities were typically assessed by an interview or content analyses. Although there is some divergence in study results depending on the measurement used, studies typically confirmed patients’ difficulty in emotional awareness and emotional theory of mind.

Several studies examined patients’ emotional awareness by analyzing the words patients used in order to describe their own and others’ emotions (Level of Emotional Awareness Scale). In one study based on this measure, patients with somatoform disorders showed reduced emotional awareness compared to healthy controls [118]. Another psychotherapy study with patients with musculoskeletal pain reported similar results. An improvement in patients’ emotional awareness from pre- to post-psychotherapy was demonstrated, particularly regarding others’ emotions. This was also accompanied by improvement in psychosomatic complaints [119]. In another study, emotional awareness in somatoform patients was found to be reduced; however, this was based only on an affect consciousness interview but not on the Level of Emotional Awareness Scale [103]. Awareness of anger, guilt, and mean negative affect was particularly diminished in the patient group in these interviews. Finally, one study examined patients’ ability to understand and reflect on their own emotions by a self-report questionnaire and found that patients with only MUS scored worse than healthy controls, although they were better in that domain than patients with a comorbid MUS and MDD or with only MDD [116].

It was suggested that patients’ reduced emotional awareness might be a function of reduced capacity to mentally represent feelings rather than a lack of emotional vocabulary [59]. The authors could support this empirically by showing that emotional awareness was correlated with emotional theory of mind in patients with conversion disorders, functional somatic syndrome, and somatoform disorders [59,118]. It was also shown that patients with fibromyalgia and somatoform disorders presented reduced emotional content and emotional theory of mind performance in their discourses compared to healthy controls [118,120]. A study investigated whether fibromyalgia patients’ such difficulty in affective theory of mind is connected to a general neuropsychological deficit. No correlation was found between affective social cognition and neuropsychological variables, suggesting that patients’ social cognition difficulties might rather be exclusively related to emotions [120]

Despite the results pointing to a reduced emotional theory of mind ability in these patients, the findings should be treated cautiously, given the diverging results between different measures of the construct. For example, one study administered an emotional theory of mind test which requires attribution of emotions based on pictures of emotional expression around the eyes and eyebrows (EYES test) [121]. This study found no difference between patients with functional motor disorders or organic movement disorders and healthy controls [122]. Similarly, when patients with conversion disorders or functional somatic syndromes and medical controls were compared, a study reported limited emotional theory of mind in SSD only in one theory of mind test, based on dynamic animations of emotions (FHAT) [123], but not in the EYES [121] test [124].

#### Emotion recognition

Patients’ abilities to recognize emotions were typically examined by computerized emotion-recognition tests. The majority of the studies confirmed reduced emotion-recognition accuracy in patients with somatoform disorders [111,125–128], fibromyalgia [120], chronic facial pain [129] and temporomandibular disorders [130], compared to healthy controls. Reduced recognition performance was reported for the individual emotions of anger, joy, sadness, fear, disgust, and neutral emotions [111,120,126,127], even after controlling for alexithymia, depression and anxiety [111]. However, in contrast to those studies, one study reported a trend towards patients’ better performance in recognition of emotions, especially of angry faces [100].

Lower emotion recognition performance was usually associated with alexithymic traits of the patients, and in some studies, the difference between patients and healthy controls diminished when alexithymia was statistically controlled. These studies suggested that emotion recognition difficulties are secondary to more basic problems, such as alexithymia or depression [127,128,130]. One interesting finding was that patients with chronic facial pain who were less accurate in recognizing emotions compared to controls were also less accurate in recognition of left/right facial movement. The authors suggested that reduced emotion recognition might not only be about emotion processing, but also about disorder-specific motor processing [129]. Only one study employed a measure involving dynamic animated emotional faces, as opposed to other static picture paradigms. This study reported no difference between patients with psychogenic non-epileptic seizures and healthy groups, indicating a possible effect of methodology on the emotion-recognition performance [131].

#### Beliefs about and attitude towards emotions

Beliefs about emotions were shown to be a predictor of SSD in five studies. For example, patients with psychogenic non-epileptic seizures reported more negative beliefs about emotions and evaluated them as more overwhelming, uncontrollable, shameful, irrational, contagious, useless, and damaging than did controls. A positive correlation was also reported between negative beliefs about emotions and seizure severity [99]. In another study, patients with chronic fatigue syndrome held significantly more beliefs about the unacceptability of experiencing and expressing emotions than controls [81,132]; especially prior to the onset of the syndrome [132]. These negative beliefs were also associated with depression, anxiety, fatigue, and self-sacrifice [133], as well as self-reported suppression [81]. It was also found that, not only patients, but also patients’ close relatives rated the patients to have more negative beliefs about emotions [132]. In a qualitative study, especially female patients emphasized their beliefs about non-acceptability of expressing negative emotions. Moreover, beliefs related to high expectations of self and social desirability was connected with how emotions are experienced [104]. Patients’ negative beliefs about emotions were shown to decrease following psychotherapy, along with other somatic and psychological improvements [133]. As opposed to negative beliefs about emotions, in one study, self-efficacy beliefs in emotion regulation were related to higher quality of life in the patients with chronic pain [134].

Other ER facets regarding attitudes towards emotions were non-acceptance of emotions, non-judging of emotions, tolerance to emotions, accessing ER strategies, and emotional clarity, which means the ability to discern how one feels. Two studies with patients with psychogenic non-epileptic seizures (PNES) examined non-acceptance of emotions and lack of emotional clarity by a commonly used self-report scale of ER difficulties. These two studies confirmed that lack of emotional clarity and not accepting emotions were significantly related to somatic complaints, emotion dysregulation, and quality of life [25,83]. One study distinguished also between PNES patients who do not respond to the external world during seizures (altered responsiveness group) and an intact responsiveness group. The study reported that the abilities to accept experienced emotions and to tolerate emotions were reduced in the altered responsiveness group compared to the intact responsiveness group, suggesting that even different semiology of the same diagnosis might show different associations with ER [135].

Another study with functional gastrointestinal disorders also showed a positive link between lack of emotional clarity and non-acceptance of emotions on the one hand, and depression on the other. These difficulties were also related to lower mindful attention [85]. Likewise, patients with functional dyspepsia reported less frequent use of acceptance compared to healthy controls [136]. Some mindfulness facets, such as non-judging of emotions and being able to describe them, were examined in patients with fibromyalgia. These variables were negatively related to alexithymia, neuroticism, and negative affect, although their association with physical health was not confirmed [86].

Differences in attitudes towards emotions among different groups of patients were also supported by a study which distinguished among patients with MUS, MUS and MDD, and only MDD and healthy controls. Patients with MUS received lower scores than healthy controls in the ability to clearly distinguish emotions, accept their emotions, and be tolerant and resilient towards emotional challenges, but higher scores than MDD or MDD and MUS patients [116].

Finally, difficulty in accessing strategies for dealing with emotions predicted anxiety and stress, as well as decreased mindful attention in functional gastrointestinal disorders [85]. This difficulty was found to be greater especially in the emotionally dysregulated patient group with psychogenic non-epileptic seizures, who had likewise more somatic symptoms, higher levels of alexithymia, and a lower quality of life [25,83].

#### Reappraisal and automatic thoughts

Three studies with patients with fibromyalgia [96,102] and chronic pain [101] failed to find a relationship between psychosomatic complaints and reappraisal (assessed with a self-report measure), especially when negative affect was statistically controlled.

On the other hand, two studies with functional neurological symptoms, one study with functional dyspepsia and another one with SSD found evidence for an association between reappraisal and SSD: Patients with functional dyspepsia reported less frequent use of positive reappraisal, but more use of rumination and other-blame compared to healthy controls [136]. One study in patients with functional neurological symptoms and another with SSD reported a lower tendency to use cognitive reappraisal compared to healthy controls [100,137]. Similarly, patients with PNES tend to utilize cognitive reappraisal to a lesser extent than controls, which was also associated with cognitive inflexibility [78]. This pattern of a less flexible cognitive style in the light of negative content was supported by another study, which found that patients with chronic tension-type headaches had more frequent automatic negative thoughts than did controls [138]. Moreover, psychotherapy with chronic pain patients increased the reappraisal capacity of these patients, accompanied by improved somatic symptom reports [87].

## Discussion

Different models and approaches to chronic somatic symptom distress agree on the central role of affective processes in the maintenance of this condition (see the reviews from [24,139]). This present review aimed at systematically investigating the existing literature with regard to the question, how patients with somatic symptom and related disorders (SSD) regulate their emotions. This was intended by 1) assessing which ER processes in SSD were examined in the body of literature, 2) scrutinizing whether relationships between ER processes, and somatic and psychological symptoms in SSD could be established and 3) whether these ER processes differ between patients with SSD, healthy controls and patients with other mental or physical disorders and finally, 4) by a comprehensive description of the characteristic ER processes of patients with SSD. Although there is a vast amount of research that has examined facets of emotion regulation in patients with SSD, to our knowledge none of the previous studies have synthesized the findings systematically. A potential reason for such a gap might be the challenge of bringing two complex and interdisciplinary concepts together: emotion regulation and SSD [10,140]. In fact, the present review ended up with diverse diagnoses and ER constructs as a consequence of the various different terms and measurement methods.

With regards to the quality of the studies, the majority met the criteria for a moderate and about thirty percent for a good quality of methods. That indicates a relatively reliable quality to have confidence in the results. Moreover, the good and moderate quality studies did not diverge in their findings and interpretations regarding the association between ER and SSD. The most commonly observed limitation was the lack of consecutive sampling, which can be explained by the difficulties in reaching patients. Almost two thirds of the studies used established diagnostic labels, and one third used broadly defined diagnoses, reflecting the disagreements in classification of SSD.

In order to summarize and synthesize the findings, we categorized the studies based on the ER constituent predominantly targeted (attention, body, or knowledge) [52]. By doing so, we sought to understand the involvement and interaction of each modality in the disturbances of emotion regulation in SSD, rather than tackling numerous ER constructs elusive to other disciplines. Such an approach promises a more precise and dynamic understanding of the patterns of emotion regulation associated with SSD, which can inform the existing theories of SSD. It thus offers a mutually comprehensible and fertile language to promote further research and an interdisciplinary bridge among disciplines handling SSD and emotion regulation (psychology, various fields of medicine, and neuropsychobiology) [41,50]. Although it would go beyond the scope of this review to finally decide on theoretical drawbacks of the conceptual distinction, a potential overlap of multiple modalities should be carefully dealt with. A combination of theory- and empirical-based approach would be useful to determine the primarily regulated emotion component.

The findings of the present review provided insightful contributions for understanding the ER processes of patients suffering from SSD in a number of ways. The findings confirm that patients with SSD encounter ER difficulties. Of particular note, the literature has quite consistently shown patients’ dysfunctional knowledge-oriented ER, such as reduced capacity of emotional awareness or emotion recognition. It was suggested that patients’ difficulties in emotional awareness might be related to their restricted ability to mentally represent emotional experiences and in higher-order cognitive-emotional processing. It was also shown that patients experience disturbances in the body- and attention-oriented ER forms, such as autonomic aberrance, muscle reactivity, negative emotional expressions, and attentional rigidity. It is also essential to draw attention to the differences between self-report and observational studies. Although patients reported to suppress their emotions to a greater extent, majority of the observational studies showed that they are actually more expressive of negative emotions, especially through bodily behavior.

It also appears noteworthy that patients suffering from different SSD, such as bodily pain, fatigue, organ-specific, functional-neurological, or gastrointestinal symptoms, presented similar ER difficulties. Disturbances such as reduced emotional awareness and reflection capacity, rigid emotional attention, or aberrant autonomic activity were shared by many different diagnoses and types of SSD. These findings are consistent with the previous research that pointed out similar non-symptom characteristics and affective difficulties shared by various diagnoses of SSD [8,13,141]. Similar ER difficulties between different diagnoses support the new DSM-5 approach to somatic symptom disorders. Despite having distinct semiology associated with the functioning of certain organs or tissues, these disorders may have ER as a common factor, which may contribute to the development or course of the disorder. It should be also kept in mind that comorbidity with other mental disorders, such as depression or anxiety might also mediate the relationship between ER and different forms of SSD. In fact, as shown in a previous study, the patient group with only medically unexplained symptoms (MUS) showed higher skills in ER compared to MUS-patients with comorbid depression and anxiety [116]. These findings can be complemented by the view that even these mental disorders may reflect rater dimensional and variably interdependent constructs than clearly distinguishable disorder entities [63].

Furthermore, experimental and diary studies lend some support to a causal role of disturbances in ER in the development and course of somatic symptoms. These studies showed that patients’ ER processes predicted and exacerbated their observed and reported somatic symptoms during experimental manipulation [80,95,97,115] or in due course [90,95,142]. Furthermore, disturbances in ER were more pronounced among patients with “medically unexplained symptoms” than among those with “medically explained” ones [25,31,94,122]. Such a difference between the two groups rules out a temporal hypothesis claiming disturbances in ER as a mere outcome of bodily distress.

The findings revealed a characteristic pattern of ER in patients with SSD. Knowledge-oriented regulation of emotions, which is related to how emotions are evaluated, made sense of, and experienced at the conscious level, seemed to be restricted in patients. The findings indicated a reduced engagement of higher-order cognitive functions with one’s own and others’ emotions, such as reduced capacities for emotional awareness, emotion recognition, and emotional theory of mind. On the other hand, studies on attention-oriented ER pointed out patients’ difficulty in disengaging their attention from the emotional material. This finding was especially supported by rather automatic attentional regulation of emotions, as measured by the Emotional Stroop test [143,144] or the *Goals* Subscale of the DERS questionnaire [82]. Patients’ bodily-oriented ER processes seemed to be concordant with the automatic attentional bias towards emotional stimuli shown in the patients. Vigilant physiological reactivity was shown by higher sympathetic activity and startle responses and difficulty in down-regulation of these parameters. The patients also presented increased impulsive behavior and negative expressions when emotionally distressed. Overall, the results suggest a course of emotion regulation among emotion components that proceed discordantly from each other. Attentional bias towards emotions and correspondingly vigilant and reactive autonomic functions, however, constrained conscious evaluation and reflection about emotions.

The relationship between somatic symptoms and discordant emotional processing through attentional, higher cognitive (knowledge), and bodily modalities was also supported by recent neuroimaging studies. Although the early research goes back to MacLean [145], only in the last two decades has this connection received attention once again. Currently, increasing findings converge to show that disintegrated processing of emotions between higher-order and subcortical structures play a role in dysregulated autonomic, immune, and HPA functioning and contribute to chronic somatic symptom [36,60,146].

The findings of this review, supported by the recent developments in neuroscience, contribute to current progress in science by moving beyond body–mind separation for understanding SSD. The results call for integrating holistic and embodied models of emotion-regulation interventions for the treatment of SSD. Treatment models working on regulation of attentional, knowledge, and bodily modalities of emotional process that go hand-in-hand can be of help. For example, in departments of psychosomatic medicine in Germany, integrative care involves body-based therapies (e.g., dance and movement therapy, Feldenkreis), creative psychotherapies (music, art), and physiotherapy, in addition to the usual individual and group psychotherapies, and these demonstrate to be effective [147]. Mindfulness-based psychotherapy that promotes mindful and non-judgmental attention to emotions [148], emotional awareness and expression therapies [119,149] have also shown patient improvement in emotion regulation and somatic symptoms. Similarly, biofeedback training aiming at altering automatic attentional and bodily processing in response to distressing stimuli might be useful. Moreover, as shown by Leong and colleagues [107] and Burns and colleagues [92] interpersonal problems associated with patients’ disturbed ER might exacerbate their distress and symptoms. Therefore, it is highly recommended that examination and intervention related to interpersonal ER should be an integral part of dealing with chronic SSD.

### Limitations and future directions

Some limitations appeared regarding the methodology and focus of the reviewed studies. Firstly, there is a scarcity of longitudinal designs, randomized controlled trials, experiments, and diary studies suited to investigate the short- and long-term causal relationship between ER and SSD. Although we could find some support for the role of ER in development of SSD, the literature is sparse and the causal mechanisms through which ER might contribute to SSD are not clear. Secondly, the measurement of ER processes is usually based on patient self-reports measured by very different questionnaires. However, assessing emotional reflection ability based on patients’ self-judgments, which are presumably disturbed, raises an inherent contradiction, which limits the validity of the findings. In fact, self-evaluations of emotional reflection and non-self-reports might measure two different things, such as explicit (conscious) and implicit (unconscious) emotional processes [150]. Thirdly, ER targeted at body and attention has received less consideration in comparison to knowledge-oriented emotion regulation. This seems to present an additional paradox when one considers the phylogenetic and ontogenetic precedence [151] of these processes, which play fundamental roles in neuro-visceral communication during ER [67]. Next, although emotion regulation is constituted and maintained by social interactions, there is a substantial lack of studies that consider the interpersonal aspects of ER in SSD. Finally, when drawing conclusions, one should take into account the publication bias towards positive results. The complex relationship between cognitive, emotional, and bodily processes of an organism embedded in its social and biological environment should be underlined. Refraining from reducing the etiology mainly to ER and psychological processes and acknowledging the role of whole biopsychosocial factors would pave the way for a better understanding of SSD.

We also acknowledge some limitations of our present review. Firstly, due to the breadth of our qualitative study goals and the heterogeneity of the reviewed studies, we did not compare the effect size of the findings. Future studies can overcome this limitation by narrowing down the specific pathways of attention-, body-, and knowledge-oriented emotion regulation. We recommend a further systematic review that statistically synthesizes the findings for the three primary ER modalities, aimed at a path analysis for prediction of SSD. Secondly, we narrowed down our emotion-related search terms only to “emotion/al regulation” and “affect regulation” but not included related search terms, such as cognitive coping, stress regulation. This might have led to missing some relevant papers that have examined emotion processing, emotional awareness, emotion recognition, cognitive emotion regulation, etc. Although these processes are accepted as regulatory by several models [41,46] including such related search terms is beyond the goals of the present manuscript, which primarily concerns investigating several diagnostic search terms considered as SSD. Thirdly, although we initially classified the studies based on objective measures (i.e. examined ER variables and diagnoses), in order to understand the mechanisms of ER in SSD, we additionally organized the studies based on components of ER, which is not an empirically established classification system. Potential drawbacks, such as overlap of multiple ER components might come into question; although it is usually the case that one distinct emotion component is primarily regulated. As a measure to this potential pitfall, we implemented a systematic method based on a combination of theory- and empirical-based approach (see the method section on organization of the studies). In addition to relying on previous studies`classification systems, we examined each items of the questionnaires, and instructions/procedures of the experiments, in order to determine the primarily examined components of ER. Fourthly, it should be kept in mind, that the studies, which examined attentional and bodily processes through questionnaires, in fact capture information about the explicit knowledge about these processes, rather than the processes themselves. This fact might result in differences in the findings of behavioral and self-report studies. In this present study, such a difference between behavioral and self-report measures was only observed in emotional expression studies. Fifthly, despite the role of feeling tone in providing a person with qualia of emotional experience, we did not include it in our analysis as it entails another corpus of extensive literature. Especially use of qualitative methods offers the potential to examine patients’ subjective affective experiences and regulation. Another limitation that should be noted is that quality appraisal items were not weighted in terms of their importance when computing a total score of quality. Finally, we only included articles in English language, which might have resulted in exclusion of valuable information coming from studies in other languages.

In conclusion, the present systematic review supported the association between ER processes and SSD. ER processes and SSD diagnoses being studied are heterogeneous. Overall, the results showed a characteristic pattern of ER in SSD, which can be distinguished by differential processing of ER constituents. More specifically, patients with SSD encounter difficulties in flexibly disengaging their (spontaneous) attention from emotional material. Comparably, bodily constituents of ER also depict an over-reactive pattern, characterized by vigilant autonomic nervous system activity, startle response, impulsive behavior, and negative emotional expressions. On the other hand, at the knowledge level, patients tend to disengage from emotional information, such as reduced identification and awareness of emotions or emotion recognition. Future research should empirically test the role of simultaneous interaction of each ER modality in SSD.

## Supporting information

S1 Checklist PRISMA 2009 checklist. (DOC)

S1 Appendix Search terms for somatic symptom and related disorders. (DOCX)

S1 File References for emotion regulation measures used in the studies. (DOCX)

S1 Table Emotion regulation variables and measurement methods in the reviewed articles. (DOCX)

S2 Table Quality ratings for articles included in the review. (DOCX)

S3 Table Diagnostic groups examined for each emotion regulation variable. (DOCX)

S4 Table Emotion regulation variables examined in each diagnostic group. (DOCX)

S5 Table Study characteristics and summaries of articles that examined attention-oriented emotion regulation. (DOCX)

S6 Table Study characteristics and summaries of articles that examined body-oriented emotion regulation. (DOCX)

S7 Table Study characteristics and summaries of articles that examined knowledge-oriented emotion regulation. (DOCX)

## Abbreviations:

SSD: Somatic Symptom and related Disorders
ER: emotion regulation
