# Supplementary material for: Emotion regulation in patients with somatic symptom and related disorders: A systematic review
Source: PLoS One. 2019 Jun 7;14(6):e0217277. doi: 10.1371/journal.pone.0217277 (PMC6555516; doi:10.1371/journal.pone.0217277)
Supplement: S1 Appendix — (DOCX) [file pone.0217277.s002.docx]

S2 Appendix

Search Terms for Somatic Symptom and Related Disorders in the Databases PubMed and PsycINFO

somatoform disorder OR somatiz* OR somatis* OR conversion disorder* OR multisomatoform OR medically unexplained* OR organically unexplained* OR psychogenic OR nonorganic OR psychosomatic syndrom* OR functional somatic syndrom* OR functional syndrom* OR functional disorder* OR functional illness* OR functional symptom* OR irritable bowel* OR functional bowel* OR functional gastrointestinal* OR functional dyspepsia* OR nonulcer dyspepsia* OR food intolerance* OR fibromyalgia* OR chronic widespread pain* OR widespread musculoskeletal pain* OR myofascial pain syndrome* OR tension-type headache* OR chronic pain* OR atypical chest pain* OR nonspecific chest pain* OR non-specific chest pain* OR atypical face pain* OR facial pain* OR chronic low back pain* OR back pain* OR panalges* OR (psychogen* AND pain) OR idiopathic pain* OR idiopathic pain disorder* OR fatigue/*psychology OR chronic fatigue syndrome* OR Fatigue Syndrome, Chronic* OR myalgic encephalomyelitis* OR myalgic encephalopathy* OR chronic epstein barr virus* OR chronic mononucleosis* OR chronic infectious mononucleosis like syndrome* OR chronic fatigue and immune dysfunction syndrome* OR effort syndrome* OR low natural killer cell syndrome* OR neuromyasthenia OR post viral fatigue syndrome* OR postviral fatigue syndrome* OR post viral syndrome* OR postviral syndrome* OR post infectious fatigue* OR postinfectious fatigue* OR royal free disease* OR royal free epidemic* OR *royal free hospital disease* OR chronic lyme disease* OR candida hypersensitivity* OR candida syndrome* OR (mitral valve prolapse* AND psychology) OR hypoglycaemia/*psychology OR sleep disorder/*psychology OR nonorganic Insomnia* OR Multiple chemical sensitivit* OR idiopathic environmental intolerance* OR electromagnetic hypersensitivity OR electrohypersensitivity OR electrosensitiv* OR IEI-EMF OR environmental illness* OR Sick Building Syndrome* OR Persian gulf syndrome OR Amalgam hypersensitivity* OR Dental Amalgam/*toxicity OR dental amalgam/*adverse effects OR silicone breast implant* OR implant intolerance* OR burning mouth* OR glossalg* OR glossodyn* OR glossopyr* OR bruxism OR temporomandibular joint disorder* OR temporomandibular disorder* OR temporomandibular joint dysfunction* OR temporomandibular joint dysfunction* OR craniomandibular disorder* OR atypical odontalgia* OR prosthesis intolerance* OR (psychogen* AND gagging) OR chronic rhinopharyngitis* OR globus syndrome* OR globus hystericus* OR hyperventilation syndrome* OR dysphonia OR aphonia OR Vertigo OR Dizziness OR repetitive strain injury *OR chronic whiplash syndrome* OR tension headache OR pseudoseizures OR hysterical seizures* OR (psychogen* AND dystonia) OR (psychogen* AND dysphagia) OR functional micturition disorder* OR functional urinary disorder* OR urethral syndrome* OR micturition dysfunction* OR (urinary retention* AND (psychogen* or psychology)) OR irritable bladder* OR painful bladder syndrome* OR interstitial cystitis* OR enuresis diurnal et nocturnal* OR anogenital syndrome* OR sexual dysfunction* OR chronic pelvic pain* OR (skin disease* AND (psychology or psychogen*)) OR (pruritus AND (psychology or psychogen* or somatoform)) OR culture-bound disorder* OR ((reduced OR impaired) AND well-being
