# Supplementary material for: Emotion regulation in patients with somatic symptom and related disorders: A systematic review
Source: PLoS One. 2019 Jun 7;14(6):e0217277. doi: 10.1371/journal.pone.0217277 (PMC6555516; doi:10.1371/journal.pone.0217277)
Supplement: S1 File — (DOCX) [file pone.0217277.s003.docx]

**S1 File. References for Emotion Regulation Measures used in the Studies**

Baer, R. A., Smith, G. T., Hopkins, J., Krietemeyer, J., & Toney, L. (2006). Using self- report assessment methods to explore facets of mindfulness. *Assessment, 13*, 27– 45.

Baron-Cohen, S., Wheelwright, S., Hill, J., Raste, Y., & Plumb, I. (2001). The “Reading the Mind in the Eyes” Test Revised Version: A study with normal adults, and adults with Asperger Syndrome or high-functioning autism. J*ournal of Child Psychology and Psychiatry, 42*(2), 241–251. doi:10.1017/s0021963001006643

Baron-Cohen, S., & Wheelwright, S. (2004). The Empathy Quotient: An investigation of adults with Asperger Syndrome or high functioning autism, and normal sex differences. *Journal of* *Autism and Developmental Disorders, 34*(2), 163–175. doi:10.1023/b:jadd.0000022607.19833.00

Bechara, A., Damasio, H., Tranel, D., & Damasio, A. R. (2005). The Iowa Gambling Task and the somatic marker hypothesis: Some questions and answers. *Trends in Cognitive Sciences, 9*(4), 159–162. doi:10.1016/j.tics.2005.02.002

Berking, M., Znoj, H. (2008). Entwicklung und Validierung eines Fragebogens zur standardisierten Selbsteinschätzung emotionaler Kompetenzen (SEK-27) (Development and validation of a self-report measure for the assessment of emotion regulation skills (SEK-27), Zeitschrift für *Psychiatrie, Psychologie und Psychotherapie, 56,* 141–153. <http://dx.doi.org/10.1024/1661-4747>.56.2.141.

Breitenstein C., Daum, I., Ackermann, H., Lütgehetmann, R., & Müller, E. (1996). Erfassung der Emotionswahrnehmung bei zentralnervosen Lasionen und Erkrankungen: Psychometrische Gutekriterien der ‘‘Tubinger Affekt Batterie’’. *Neurologie & Rehabilitation, 2*(9).

Brown, K.W. & Ryan, R.M. (2003). The benefits of being present: Mindfulness and its role in psychological well-being. *Journal of Personality and Social Psychology, 84,* 822–848.

Burns, J. W., Quartana, P., & Bruehl, S. (2011). Anger suppression and subsequent pain behaviors among chronic low back pain patients: Moderating effects of anger regulation style. *Annals of* *Behavioral Medicine, 42*(1), 42–54. doi: 10.1007/s12160-011-9270-4

Constantinou, E., Van Den Houte, M., Bogaerts, K., Van Diest, I., & Van den Bergh, O. (2014). Can words heal? Using affect labeling to reduce the effects of unpleasant cues on symptom reporting. *Frontiers in Psychology, 5.* doi:10.3389/fpsyg.2014.00807

Elderen, T., Maes, S., Komproe, I., & Kamp, L. (1997). The development of an anger expression and control scale. *British Journal of Health Psychology, 2*(3), 269–281. doi:10.1111/j.2044-8287.1997.tb00541.x

Friesen, W., Ekman, P. (1983). *EMFACS-7:* *Emotional Facial Action Coding System.* Unpublished manual, University of California.

Froming, K. B., Levy, C. M., Ekman, P. (2004): *CATS – Comprehensive Affect Testing System,* Brief German version.

Garnefski, N., Kraaij, V. (2006). Cognitive Emotion Regulation Questionnaire – Development of a short 18‑item version (CERQ‑short). *Personality and Individual Differences*, *41*,1045–1053.

Gottman, J. M., McCoy, K., Coan, J., & Collier, H. (1995). *The Specific Affect Coding System* *(SPAFF) for observing emotional communication in marital and family interaction.* Mahwah, NJ: Erlbaum.

Gratz, K. L., & Roemer, L. (2004). Multidimensional assessment of emotion regulation and dysregulation: Development, factor structure, and initial validation of the difficulties in emotion regulation scale. *Journal of Psychopathology and Behavioral Assessment, 26*(1), 41–54. doi:10.1023/b:joba.0000007455.08539.94

Gross, J. J., & John, O. P. (2003). Individual differences in two emotion regulation processes: Implications for affect, relationships, and well-being. *Journal of Personality and Social* *Psychology, 85*(2), 348–362. doi:10.1037/0022-3514.85.2.348

Hayes, S. C., Strosahl, K. D., Wilson, K. G., Bissett, R. T., Pistorello, J., Toarmino, D. … & McCurry, S. M. (2004). Measuring experiential avoidance: A preliminary test of a working model. *The Psychological Record, 54,* 553–578.

Hofmann, S. G., & Kashdan, T. B. (2009). The Affective Style Questionnaire: Development and psychometric properties. *Journal of Psychopathology and Behavioral Assessment, 32*(2), 255–263. doi:10.1007/s10862-009-9142-4

Hollon, S. D., & Kendall, P. C. (1980). Cognitive self-statements in depression: Development of an Automatic Thoughts Questionnaire. *Cognitive Therapy and Research, 4,* 383–395.

Kessler, H., Bayerl, P., Deighton, R., & Traue, H.C. (2002). Facially expressed emotion labeling (FEEL): PC-gestutzter Test zur Emotionserkennung. *Verhaltenstherapie und* *Verhaltensmedizin, 23*, 297–306.

Lane, R., Quinlan, D., Schwartz, G., Walker, P., & Zeitlin, S. (1990). The Levels of Emotional Awareness Scale: A cognitive-developmental measure of emotion. *Journal of Personality* *Assessment, 55*(1), 124–134. doi:10.1207/s15327752jpa5501&2_12

Lundqvist, D., Flykt, A., & Öhman, A. (1998). *The Karolinska Directed Emotional Faces - KDEF*, CD ROM from Department of Clinical Neuroscience, Psychology section, Karolinska Institutet, ISBN 91-630-7164-9.

Manser, R., Cooper, M., & Trefusis, J. (2011). Beliefs about emotions as a metacognitive construct: Initial development of a self-report questionnaire measure and preliminary investigation in relation to emotion regulation. *Clinical Psychology & Psychotherapy, 19*(3), 235–246. doi:10.1002/cpp.745

Monsen, J. T., Eilertsen, D. E., Melgård, T., & Ødegård, P. (1996). Affects and affect consciousness: Initial experiences with the assessment of affect integration. *The Journal of* *Psychotherapy Practice and Research, 5*(3), 238–249.

Rimes, K. A., & Chalder, T. (2010). The Beliefs about Emotions Scale: Validity, reliability and sensitivity to change. *Journal of Psychosomatic Research, 68*(3), 285–292. doi:10.1016/j.jpsychores.2009.09.014

Salovey, P., Mayer, J. D., Goldman, S. L., Turvey, C., & Palfai, T. P. (1995). Emotional attention, clarity, and repair: Exploring emotional intelligence using the Trait Meta-Mood Scale. *Emotion, Disclosure, & Health.,* 125–154. doi:10.1037/10182-006

Schönenberg, M., Mares, L., Smolka, R., Jusyte, A., Zipfel, S., & Hautzinger, M. (2014). Facial affect perception and mentalizing abilities in female patients with persistent somatoform pain disorder. *European Journal of Pain, 18*(7), 949–956. doi:10.1002/j.1532-2149.2013.00440.x

Schutte, N.S., Malouff, J.M., & Bhullar, N. (2009). The Assessing Emotions Scale. In C. Stough, D. Saklofske, & J. Parker (Eds.), *The assessment of emotional intelligence.* New York: Springer Publishing, 119–135.

Spielberger, C.D. (1998). *Manual for the State–Trait Anger Expression Inventory (STAXI).* Odessa, FL: Psychological Assessment Resources.

Watson, M., & Greer, S. (1983). Development of a questionnaire measure of emotional control. *Journal of Psychosomatic Research, 27*(4), 299–305. doi:10.1016/0022-3999(83)90052-1

White, S. J., Coniston, D., Rogers, R., & Frith, U. (2011). Developing the Frith-Happé animations: A quick and objective test of Theory of Mind for adults with autism. *Autism Research, 4*(2), 149–154. doi:10.1002/aur.174
