# Supplementary material for: Emotion regulation in patients with somatic symptom and related disorders: A systematic review
Source: PLoS One. 2019 Jun 7;14(6):e0217277. doi: 10.1371/journal.pone.0217277 (PMC6555516; doi:10.1371/journal.pone.0217277)
Supplement: S1 Table — (DOCX) [file pone.0217277.s004.docx]

**S1 Table.** **Emotion Regulation Variables and Measurement Methods in the Reviewed Articles**

| **ER component** | **ER variable** | **Measurement** |
| --- | --- | --- |
| **Attention** | Acting with awareness | Five Facet Mindfulness Questionnaire, Actaware Subscale (e.g., ‘‘I find myself doing things without paying attention’’) (Baer, Smith, Hopkins, Krietemeyer, & Toney, 2006). |
|  | Attention switching, flexibility, fixedness and attentional hyper-vigilance | Emotional Stroop Task, Task Switching Paradigm (attentional bias to emotional material). |
|  | Attending to emotions | Difficulties in Emotion Regulation Scale, Awareness Subscale (*e.g.* e.g., “I pay attention to how I feel”) (Gratz & Roemer, 2004).  Emotion Regulation Skills Questionnaire, Awareness Subscale (e.g., “I paid attention to my feelings”) (Berking & Znöj, 2008). |
|  | Difficulties engaging in goal directed behavior | Difficulties in Emotion Regulation Scale, Goals Subscale (e.g., “When I’m upset, I have difficulty focusing on other things”) (Gratz & Roemer, 2004). |
|  | Mindful attention | Mindful Attention Awareness Scale (e.g., “I could be experiencing some emotion and not be conscious of it until sometime later”) (Brown & Ryan, 2003). |
|  | Observing, noticing, and attending to sensations, thoughts, and feelings | Five Facet Mindfulness Questionnaire, Observe Subscale (e.g., “I pay attention to how my emotions affect my thoughts and behavior”) (Baer et al., 2006). |
|  | Suppression of thoughts and feelings | Experimental paradigm (subjects were instructed to suppress what they were thinking and feeling about the task, through an “ironic effect,” greater attention deployment). (Burns et al., 2011). |
| **Body** | Action readiness to confront distressing situations  Affect-modulated startle in the muscles | Emotion Regulation Skills Questionnaire, Readiness to Confront Subscale (e.g., “I did what I had planned, even if it made me feel uncomfortable or anxious” (Berking & Znöj, 2008).  Electromyogram |
|  | Anger expression and suppression | Diary sampling  Self-Expression and Control Scale (Elderen, Maes, Komproe, & Kamp, 1997) |
|  |  | State-Trait Anger Expression Inventory (Spielberger, 1988) |
|  | Autonomic nervous system activity | Blood pressure, heart rate, heart rate variability, skin conductance response, respiration rate, Electromyogram, cortisol levels, respiratory sinus arrhythmia |
|  | Behavioral expression of validation and invalidation | Specific Affect Coding System (Gottman, McCoy, Coan, & Collier, 1995). |
|  | Control of emotional reactions  Distinguishing bodily sensations during emotions  Emotional behavior | Courtauld Emotional Control Scale (e.g., “I let others see how I feel”) (Watson & Greer, 1983).  Emotion Regulation Skills Questionnaire, Sensations Subscale (e.g., “My physical sensations were a good indication of how I was feeling”) (Berking & Znöj, 2008). |
|  | Emotional decision making | IOWA Gambling Task (requires awareness of internal bodily signals during affective arousal) (Bechara, Damasio, Tranel, & Damasio, 2005). |
|  | Expressive suppression | Emotion Regulation Questionnaire, Suppression Subscale (e.g., “I control my emotions by not expressing them”) (Gross & John, 2003). |
|  | Facial emotional expression | Emotional Facial Action Coding System (Friesen & Ekman, 1983). |
|  | Impulse control difficulties | Difficulties in Emotion Regulation Scale (Impulse Subscale; e.g., “When I’m upset, I lose control over my behaviors”) (Gratz & Roemer, 2004). |
|  | Nonverbal expression of emotions | Affect Consciousness Interview (Monsen, Eilertsen, Melgård, & Ødegård, 1996). |
|  | Progressive muscle relaxation | Experimental instruction |
| **Knowledge** | Access to emotion regulation strategies | The Difficulties in Emotion Regulation Scale, Strategies Subscale (e.g., “When I’m upset, I believe that I’ll end up feeling very depressed” (Gratz & Roemer, 2004). |
|  | Acceptance of emotions  Adjusting emotional experiences | Acceptance and Action Questionnaire (e.g., “I’m afraid of my feelings; emotions cause problems in my life”) (Hayes et al., 2004).  Emotion Regulation Skills Questionnaire, Acceptance Subscale (e.g., “I accepted my emotions”) (Berking & Znöj, 2008).  Cognitive Emotion Regulation Questionnaire (“e.g., I think that I have to accept that this has happened”) (Garnefski & Kraaij, 2006).  Affective Style Questionnaire, Adjust Subscale (e.g., “I can get into a better mood quite easily”) (Hofmann & Kashdan, 2010). |
|  | Affective memory | Experimental Affective Memory Performance Test |
|  | Automatic negative thoughts | Automatic Thoughts Questionnaire (Hollon & Kendall, 1980). |
|  | Beliefs about emotions | Beliefs about Emotions Scale (Rimes & Chalder, 2010), Beliefs About Emotions Questionnaire (Manser, Cooper, & Trefusis, 2011). |
|  | Describing emotional experience | Five Facet Mindfulness Questionnaire, Describe Subscale (e.g., “I’m good at finding words to describe my feelings”) (Baer et al., 2006). |
|  | Efficacy in emotional regulation | Assessing Emotions Scale (e.g., “I am aware of the non-verbal messages I send others”) (Schutte, Malouff, & Bhullar, 2009) |
|  | Emotional clarity | The Difficulties in Emotion Regulation Scale, Clarity Subscale (e.g., “I have difficulty making sense out of my feelings”) (Gratz & Roemer, 2004), Trait Meta-Mood Scale (Salovey et al.,1995).  Emotion Regulation Skills Questionnaire, Clarity Subscale (e.g., “I was clear about what emotions I was experiencing”) (Berking & Znöj, 2008). |
|  | Emotional awareness for self & other | The Levels of Emotional Awareness Scale (Lane, Quinlan, Schwartz, Walker, & Zeitlin, 1990). The Affect Consciousness Interview (Monsen et al., 1996), Assessing Emotions Scale, Appraisal Subscale (Schutte et al., 2009) |
|  | Emotion recognition | Karolinska Directed Emotional Faces Battery (Lundqvist, Flykt, & Öhman, 1998), Tübinger Affekt Batterie (Breitenstein, Daum, Ackermann, Lutgehetmann, & Muller, 1996), Facially Expressed Emotion Labeling (Kessler, Bayerl, Deighton, & Traue, 2002), Animated Morphing Paradigm (Schönenberg et al., 2014), Comprehensive Affect Testing System (Froming, Levy, Ekman, 2004). |
|  | Emotional theory of mind | Emotional content in Frith-Happé-Animations Task (White et al., 2011), Reading the Mind in the Eyes Test (Baron-Cohen et al., 2001) |
|  | Empathy capacity | Empathy Quotient (Baron-Cohen & Wheelwright, 2004). |
|  | Modification of emotional experience  Non-acceptance of emotional responses | Emotion Regulation Skills Questionnaire, Modification Subscale (e.g., “I was able to inﬂuence my negative feelings”) (Berking & Znöj, 2008).  The Difficulties in Emotion Regulation Scale, Non-acceptance Subscale (e.g., “When I’m upset, I feel guilty for feeling that way”) (Gratz & Roemer, 2004) |
|  | Non-judging of emotions & thoughts | Five Facet Mindfulness Questionnaire, Non-judge Subscale (e.g., “I criticize myself for having irrational or inappropriate emotions”) (Baer et al., 2006). |
|  | Other-blame  Reappraisal  Rumination  Self-support in emotional challenges  Tolerance and resilience to emotional confrontations  Understanding emotions | Cognitive Emotion Regulation Questionnaire (e.g., *“*I feel that others are to blame for it”) (Garnefski & Kraaij, 2006).  Emotion Regulation Questionnaire (Gross & John, 2003)  Cognitive Emotion Regulation Questionnaire (e.g., *“*I think I can learn something from the situation”) (Garnefski &Kraaij, 2006).  Cognitive Emotion Regulation Questionnaire (e.g., *“*I often think about how I feel about what I have experienced”) (Garnefski &Kraaij, 2006).  Emotion Regulation Skills Questionnaire, Self-support Subscale (e.g., “I supported myself in emotionally distressing situations”) (Berking & Znöj, 2008).  Emotion Regulation Skills Questionnaire, Tolerance Subscale (e.g., “I felt I could tolerate my negative feelings”) (Berking & Znöj, 2008).  Affective Style Questionnaire, Adjust Subscale (e.g., “I can tolerate being upset”) (Hofmann & Kashdan, 2010).  Emotion Regulation Skills Questionnaire, Understanding Subscale (e.g., “I was aware of why I felt the way I felt”) (Berking & Znöj, 2008). |
|  | Utilization of emotions | Assessing Emotions Scale (e.g., “When my mood changes, I see new possibilities”) (Schutte et al., 2009). |
