# Supplementary material for: Emotion regulation in patients with somatic symptom and related disorders: A systematic review
Source: PLoS One. 2019 Jun 7;14(6):e0217277. doi: 10.1371/journal.pone.0217277 (PMC6555516; doi:10.1371/journal.pone.0217277)
Supplement: S2 Table — (DOCX) [file pone.0217277.s005.docx]

# S2 Table. Quality Ratings of the Studies Included in the Review

| **Authors** | **Specificity of examined diagnoses** | **Design** | **Inclusion exclusion criteria** | **Established (research) diagnostic criteria (RDC)** | **Comparison groups** | **Comparison groups matched?** | **Sample type / recruitment mode** | **Sample size** | **Use of validated instruments** | **Quality of statistics** | **Total quality score** |
| --- | --- | --- | --- | --- | --- | --- | --- | --- | --- | --- | --- |
|  | **Diagnostic category broad / unspecific vs. narrow** | **Patient reported outcomes (PRO) or multilevel** | **Provided information sufficient? (yes/no)** | **Not available n.a.; no criteria stated; criteria applied** | **None; healthy controls (HC); other medical condition (OMC)** | **Demographic match (yes/no)** | **Consecutive sampling? (yes/no)** | **Based on effect size consideration (Brown & Reuber, 2016)** | **For primary and secondary outcomes (yes/no)** | **Inferential (yes/no) ^a^** | **+ 25-49%, ++ 50-79%,  +++ 80% or more** |
| Agar-Wilson & Jackson (2012) | broad | PRO | no | - (n.a.) | none | - | no | large | yes | yes | ++ (2 of 4) |
| Baslet (2017) | specific | PRO | no | - (n.a.) | none | - | no | large | yes | no | + (1 of 4) |
| Beck et al., (2013) | specific | multilevel | no | yes (ICD) | HC | no | no | moderate | yes | no | + (2 of 6) |
| Brooks et al., (2017) | specific | PRO | no | yes (Oxford or CDC) | HC | yes | no | large | yes | no | ++ (3 of 6) |
| Brown et al. (2013) | specific | PRO | yes | - (n.a.) | OMC | no | yes | small | yes | yes | +++ (4 of 5) |
| Bruehl et al., 2007 | broad | multilevel | yes | - (n.a.) | HC | no ^b^ | no | very small | yes | yes | ++ (3 of 5) |
| Bruehl et al., (2012) | broad | multilevel | yes | - (n.a.) | HC | no | yes (sample was part of a larger study) | moderate | yes | yes | +++ (4 of 5) |
| Burger et al., (2016) | broad | PRO | yes | - (n.a.) | none | - | yes | large | yes | no | ++ (3 of 4) |
| Burns et al., (2015)* | broad | multilevel | yes | - (n.a.) | HC/OMC | no | no | large | yes | yes | ++ (3 of 5) |
| Burns et al., (2008) | broad | multilevel | yes | - (n.a.) | none | - | no | large | yes | yes | ++ (3 of 4) |
| Burns et al., (2011) | broad | PRO | yes | - (n.a.) | none | - | no | moderate | yes | yes | ++ (3 of 4) |
| Castelli et al. (2013) | specific | PRO | yes | yes (American Academy of Orofacial Pain) | HC | no ^b^ | yes | moderate | yes | no | ++ (4 of 6) |
| Chavooshi et al. (2016) | broad | PRO | yes | - (n.a.) | none | - | no | large | yes | no | ++ (2 of 4) |
| Constantinou et al., (2014) | specific | multilevel | no | yes (Rome III) | HC | no ^b^ | no | moderate | yes | yes | ++ (3 of 6) |
| De Greck et al., (2011) | specific | multilevel | no | yes (DSM IV) | HC | yes | no | small | yes | no | ++ (3 of 6) |
| Del Rio-Casanova et al. (2018) | specific | PRO | yes | yes (DSM-V) | HC | no | yes | moderate | yes | yes | +++ (5 of 6) |
| Demartini et al., (2014) | specific | PRO | yes | - (n.a.) | OMC; HC | yes | no | moderate | yes | no | ++ (3 of 5) |
| Di Tella et al., (2015) | specific | multilevel | yes | no | HC | yes | yes | moderate | yes | no | ++ (4 of 6) |
| Elsenbruch et al (2010) | specific | multilevel | yes | yes (Rome III) | HC | no | no | very small | yes | yes | ++ (4 of 6) |
| Erkic et al., (2017) | broad | multilevel | yes | Yes (DSM IV or V) | HC | yes | no | moderate | yes | yes | +++ (5 of 6) |
| Fournier et al. (2018) | specific | multilevel | yes | yes (Rome II) | HC | no | no | small | yes | no | ++ (3 of 6) |
| Geenen et al., (2012) | specific | PRO | no | yes (ACR) | none | - | no | large | yes | yes | ++ (3 of 5) |
| Gul & Ahmad (2014) | specific | multilevel | yes | yes (DSM IV) | HC | yes | no | large | yes | yes | +++ (5 of 6) |
| Haas et al. (2013) | specific | multilevel | yes | yes (RDC TMD) | HC | yes | yes | small | yes | no | +++ (5 of 6) |
| Kienle et al. (2018) | broad | multilevel | yes | yes (ICD) | HC | no^b^ | no | small | yes | yes | ++ (4 of 6) |
| Kilkens et al., (2004) | specific | multilevel | yes | yes (Rome) | HC | yes | no | very small | yes | yes | +++ (5 of 6) |
| Kleiman et al., (2016) | broad | multilevel | yes | yes (ICD) | None | - | no | moderate | yes | yes | +++ (4 of 5) |
| Koenig et al., (2015) | specific | multilevel | no | yes (WAD I-III) | HC | no | no | moderate | yes | no | + (2 of 6) |
| Leong et al., (2011) | broad | multilevel | yes | - (n.a.) | HC | no | no | large | yes | yes | ++ (3 of 5) |
| Mazaheri (2015) | specific | PRO | no | yes (Rome III) | none | - | yes ^c^ | large | yes | yes | +++ (4 of 5) |
| Mazaheri et al. (2016) | specific | PRO | no | yes (Rome III) | HC | yes | no | moderate | yes | no | ++ (3 of 6) |
| Merten & Brunnhuber (2004) | broad | multilevel | no | yes (DSM-IV) | HC | no | no | very small | yes | no | + (2 of 6) |
| Ozturk et al., (2016) | specific | multilevel | yes | yes (DSM V) | HC | yes | yes | moderate | yes | yes | +++ (6 of 6) |
| Pedrosa Gil et al., (2008) | specific | multilevel | yes | yes (ICD) | HC | yes | no | small | yes | no | ++ (4 of 6) |
| Pollatos, Dietel et al. (2011)^**^ | specific | multilevel | yes | yes (Kroenke) | HC | yes | no | small | yes | yes | +++ (5 of 6) |
| Rasting et al., (2005) | broad | multilevel | no | no | none | - | no | very small | yes | no | + (1 of 4) |
| Rimes & Chalder (2010) | specific | PRO | no | yes (Oxford) | HC | no | no | large | yes | no | + (2 of 6) |
| Rimes et al., (2016) | Specific | multilevel | no | Yes (CDC) | HC | no | no | large | yes | yes | ++ (3 of 6) |
| Roberts et al. (2012) | specific | multilevel | yes | - (n.a.) | HC | no ^b^ | no | small | yes | no | + (2 of 5) |
| Sayar et al., (2004) | specific | PRO | yes | yes (ACR) | OMC; HC | no | yes | small | yes | yes | +++ (5 of 6) |
| Schoenenberg et al., (2015) | specific | multilevel | no | - (n.a.) | HC | yes | yes | very small | yes | no | ++ (3 of 5) |
| Schwarz et al., (2017) | specific | PRO | yes | yes (DSM-V) | HC; OMCs | no | no | large | yes | yes | ++ (4 of 6) |
| Seignourel et al., (2007) | specific | multilevel | yes | - (n.a.) | HC | yes | no | very small | yes | yes | +++ (4 of 5) |
| Sibelli et al., (2017) | specific | PRO | no | yes | n.a. | n.a. | no | moderate | no^e^ | n.a. | n.a. |
| Steffen et al. (2015) | broad | PRO | yes | yes (ICD) | HC | no ^b^ | no | moderate ^d^ | yes | yes | ++ (4 of 6) |
| Stonnington, et al., (2013)*** | specific | multilevel | yes | yes (DSM IV) | OMC | yes | yes | moderate | yes | yes | +++ (6 of 6) |
| Subic-Wrana et al., (2010) | broad | multilevel | no | yes (ICD) | HC | yes | yes | moderate | yes | no | ++ (4 of 6) |
| Twiss et al. (2009) | specific | multilevel | yes | yes (Rome II) | HC | no | no | very small | yes | yes | ++ (4 of 6) |
| Uliaszek et al., (2012) | specific | multilevel | no | - (n.a.) | none | - | yes | moderate | yes | no | ++ (2 of 4) |
| Urbanek et al. (2014) | specific | PRO | yes | - (n.a.) | HC | no | no | moderate ^d^ | yes | yes | ++ (3 of 5) |
| van Middendorp, et al., (2008) | specific | PRO | no | yes (ACR) | HC | no | no | large | yes | yes | ++ (3 of 6) |
| van Middendorp et al., (2010) | specific | PRO | yes | yes (ACR) | none | - | no | large | yes | yes | +++ (4 of 5) |
| Veehof et al., (2011) | specific | PRO | no | no | none |  | no | large | yes | no | + (1 of 4) |
| von Piekartz, et al., (2015) | broad | multilevel | yes | yes (RDC TMD) | HC | yes | no | small | yes | yes | +++ (5 of 6) |
| Waller & Scheidt (2004) | specific | multilevel | yes | yes (ICD) | HC | yes | no | small | yes | yes | +++ (5 of 6) |
| Walteros et al. (2011) | specific | multilevel | no | no | HC | no | no | very small | yes | no | - (1 of 6) |
| Wingenfeld (2011) | broad | multilevel | yes | yes (DSM IV) | HC | no ^b^ | no | moderate | yes | no | ++ (3 of 6) |
| Wong & Fielding (2013) | broad | PRO | yes | - (n.a.) | none | - | no | large | yes | yes | ++ (3 of 4) |
| Yucel et al. (2002) | specific | PRO | yes | yes (IHSC) | HC | yes | yes | large | yes | no | +++ (5 of 6) |
| Zautra et al., (2001) | specific | PRO | no | yes (ACR) | OMC | No | no | large | yes | no | + (2 of 6) |
| Zoccali et al., (2006) | specific | PRO | no | yes (Rome II) | HC | yes | no | moderate | yes | no | ++ (3 of 6) |

*identical sample with that of Burns et al., (2016).

^**^identical sample with that of Pollatos, Herbert et al., (2011).

***identical sample with that of Lane et al., (2015).

^a^ inferential (models controlling for potential confounders; information for probability distribution provided), as opposed to descriptive/ explorative (simple group comparisons, correlations)

^b^ authors report a balanced recruitment mode or provide post-hoc sensitivity analyses

^c^ census method applied

^d^ (power analysis provided)

^e^ data driven based on grounded research principles, qualitative study
