# Supplementary material for: Emotion regulation in patients with somatic symptom and related disorders: A systematic review
Source: PLoS One. 2019 Jun 7;14(6):e0217277. doi: 10.1371/journal.pone.0217277 (PMC6555516; doi:10.1371/journal.pone.0217277)
Supplement: S3 Table — (DOCX) [file pone.0217277.s006.docx]

**S3 Table. Diagnostic Groups Examined for each Emotion Regulation Variable**

| **Classification** | **ER Variables** | **ER Measures** |
| --- | --- | --- |
| **Chronic pain** | 1. Expressive suppression  (Chavooshi, et al., 2016^1^) 2. Emotional expression  (Leong, Cano & Johansen, 2011^2^; Merten & Brunnhuber, 2004^3^) 3. Reappraisal (Chavooshi, et al., 2016^1^; Wong & Fielding, 2013^2^) 4. Efficacy in emotion regulation, awareness, and utilization of emotions (Agar-Wilson & Jackson, 2012^4^) | 1. Emotion Regulation Questionnaire 2. The Specific Affect Coding System 3. Emotional Facial Action Coding System 4. Assessing Emotions Scale (Efficacy, Appraisal and Utilization Subscales) |
| **Chronic low back pain** | 1. Anger expression style  (Bruehl, et al., 2007^1^, 2012^1^; Burns, et al., 2008^1^, 2011^1^) 2. Real time anger expression vs. inhibition (Burns, et al., 2008^2^) 3. Emotional thought suppression  (Burns, et al., 2011^3^) 4. Momentary daily anger expression and inhibition (Bruehl, et al., 2012^4^; Burns, Gerhart, et al., 2015^4^ & 2016^4^) | 1. Anger Expression Inventory 2. Experimental manipulation of anger expression 3. Experimental manipulation through thought suppression 4. Electronic diary assessment |
| **Chronic musculoskeletal pain** | 1. Emotional awareness & emotional theory of mind (Burger et al., 2016^1^) | 1. Level of Emotional Awareness Scale |
| **Medically unexplained pain** | 1. Mindful attention (Chavooshi, et al., 2016^1^) | 1. Mindful Attention Awareness Scale |
| **Persistent somatoform pain disorder** | 1. Autonomic nervous system activity (Kleiman. et al., 2016^1,2,3^) | 1. Heart Rate 2. Skin Conductance Response 3. Electromyogram |
| **Fibromyalgia** | 1. Attending to emotions (Veehof, et al., 2011^4^) 2. Anger expression style  (Sayar, et al., 2004^1^; van Middendorp, et al., 2008^2^, 2010^2^) 3. General emotional expression (Geenen, et al., 2012^10^; van Middendorp, et al., 2008^11^) 4. Emotional decision making  (Walteros, et al., 2011^8^) 5. Acceptance & non-judgement of emotions (Veehof, et al, 2011^3^) 6. Emotion recognition, empathy and emotional theory of mind (Di Tella, 2015^5,6,7^) 7. Emotional clarity (Zautra, et al., 2001^9^) 8. Reapparaisal (Geenen, et al., 2012^11^; van Middendorp, et al., 2008^11^) | 1. State Trait Anger Expression Inventory 2. Self-Expression and Control Scale 3. Five Facet Mindfulness Questionnaire (Non-judge & describe subscales) 4. Five Facet Mindfulness Questionnaire (Observe subscale) 5. Reading the Mind in the Eyes Test 6. Empathy Quatient 7. Ekman 60 8. Iowa Gambling Task 9. Trait Meta-Mood Scale 10. Emotional Approach Coding Scale 11. Emotion Regulation Questionnaire |
| **Myofascial pain** | 1. Anger expression style   (Castelli, et al., 20131) | 1. State Trait Anger Expression Inventory |
| **Chronic whiplash-**  **associated**  **disorders** | 1. Autonomic nervous system activity (Koenig, et al., 2015^1^) | 1. Heart Rate Variability |
| **Temporomandibular disorders** | 1. Emotion recognition   (Haas, et al., 2013^1^) | 1. Facially Expressed Emotion Labeling |
| **Chronic facial pain** | 1. Emotion recognition   (Piekarzt, et al., 2015^1^) | 1. Facially Expressed Emotion Labeling |
| **Tension-type headache** | 1. Automatic negative thoughts   (Yucel, et al., 2002^1^) | 1. Automatic Thoughts Scale |
| **Chronic fatigue syndrome** | 1. Belifes about acceptability of emotions   (Rimes & Chalder, 2010^1^) | 1. Beliefs about Emotions Scale |
| **Functional abdominal pain** | 1. Autonomic nervous system activity   (Walker, et al., 2017^1, 2^) | 1. Heart rate 2. Heart rate variability |
| **Functional gastrointestinal disorders** | 1. Acceptance and clarity of emotions 2. Attending to emotions 3. Mindful attention 4. Engaging in goal directed behaviour 5. Impulse control difficulties   (Mazaheri, 2015) | 1. Difficulties in Emotion Regulation Scale (DERS) (Clarity, Strategies and Accept) 2. DERS-Awareness 3. Mindful Attention Awareness Scale 4. DERS-Goals 5. DERS-Impulse |
| **Functional dyspepsia** | 1. Emotional acceptance (Mazaheri, et al., 2016^1^) 2. Positive reappraisal, blaming & rumination   (Mazaheri, et al., 2016^2^) | 1. Emotion Regulation Skills Questionnaire 2. Cognitive Emotion Regulation Questionnaire |
| **Interstitial**  **cystitis/painful**  **bladder syndrome** | 1. Affect-modulated startle   (Twiss, et al., 2009^1^) | 1. Affect modulated Startle in the Eye-Blink |
| **Irritable bowel syndrome** | 1. Anger expression style   (Zoccali, et al., 2006^1^)   1. Muscle relaxation   (Elsenbruch, et al., 2010^2^)   1. Emotional Expression (Fournier, et al., 2018^5^) 2. Autonomic nervous system (Fournier, et al., 2018^7,8,9^) 3. Emotional exposure and emotion labelling   (Constantinou, et al., 2014^3^)   1. Affective memory   (Kilkens, et al., 2004^4^). | 1. State Trait Anger Expression Inventory 2. Manipulation of Muscle Relaxation 3. Modified Affect Labeling Task 4. Affective Memory Performance Test 5. Emotional Facial Action Coding System 6. Heart rate 7. Heart rate variability, 8. Cortisol level |
| **Medically unexplained symptoms** | 1. Emotional understanding 2. Modification & self-support during emotional challenges 3. Attending to emotions 4. Action readiness to confront emotions 5. Distinguishing bodily sensations   (Schwarz, et al., 2017^1^) | 1. Emotion Regulation Skills Questionnaire |
| **Somatoform disorders/ Somatic symptom disorders/ Multisomatoform disorders/** | 1. Autonomic nervous system activity   (Pollatos Dietel, et al., 2011^1,2,3,4^; Pollatos. Herbert, et al., 2011^1,2,3^)   1. Non-verbal expression of emotions (Waller & Scheidt, 2004^7^) 2. Emotional awareness & emotional theory of mind   (Subic-Wrana, et al., 2010^5,6^; Waller & Scheidt, 2004^5,7^)   1. Emotion recognition   (Beck, et al., 2013^8^, De Greck, et al., 2011^9^, Ozturk, et al., 2016^10^; Pedrosa Gil, et al., 2008^11^, Pollatos, Herbert et al., 2011^12^) | 1. Heart Rate Variability 2. Heart Rate 3. Skin Conductance Response 4. Respiration Rate 5. Level of Emotional Awareness Scale 6. Emotional Content in Frith-Happe-Animations Task 7. Affect Consciousness Interview 8. Comprehensive Affect Testing System 9. Tübinger Affekt Batterie 10. Ekman & Friesen faces 11. Facially Expressed Emotion Labeling 12. Karolinska Directed Emotional Faces battery |
| **Psychosomatic disorders** | 1. Attention switching   (Wingenfeld, et al., 2011^1^)   1. Facial emotional expression   (Rasting, Brosig, & Beutel, 2005^2^) | 1. Emotional Stroop Test 2. Emotional Facial Action Coding System |
| **Functional**  **neurological**  **symptoms** | 1. Expressive suppression   (Steffen, et al., 2015^1^)   1. Reappraisal (Kienle, et al., 2018^1^) | 1. Emotion Regulation Questionnaire |
| **Psychogenic non-**  **epileptic seizures** | 1. Attention switching   (Gul & Ahmad, 2014^1^)   1. Attending to emotions   (Brown, 2011^2^; Uliaszek, Prensky & Baslet, 2012^2^)   1. Engaging in goal directed behaviour (Brown, 2011^3^; Uliaszek, et al., 2012^3^) 2. Control of emotional reactions (Urbanek, et al., 2014^4^) 3. Expressive suppression   (Gul & Ahmad, 2014^5^)   1. Impulse control difficulties   (Brown, et al., 2013^6^; Uliaszek, et al., 2012^6^)   1. Positive & negative emotional behavior (Roberts, et al., 2012^12^) 2. Autonomic nervous system activity (Roberts, et al., 2012^13,14^) 3. Emotion recognition   (Schoenenberg, et al., 2015^7^)   1. Acceptance and clarity of emotions (Brown, 2011^8^; Uliaszek, et al., 2012^8^, Urbanek, et al., 2014^8^) 2. Reappraisal (Gul & Ahmad, 2014^9^) 3. Toleration and acceptance of emotions (Baslet, et al., 2017^10,11^) | 1. Task switching paradigm 2. Difficulties in Emotion Regulation Scale (DERS)-Awareness 3. DERS-Goals 4. Cortauld Emotional Control Scale 5. Emotion Regulation Questionnaire-Suppression 6. DERS-Impulse 7. Animated morphing paradigm 8. DERS- Clarity, Strategies and Accept 9. Emotion Regulation Questionnaire-Reappraisal 10. Acceptance and Action Questionnaire 11. Affective Style Questionnaire 12. Observation coding 13. Heart rate 14. Respiratory sinus arithymia |
| **Psychogenic**  **movement**  **disorders** | 1. Affect modulated startle   (Seignourel, et al., 2007^1^) | 1. Affect modulated Startle in the Eye-Blink (EMG) |
| **Conversion disorders &/vs. functional somatic syndromes** | 1. Emotional awareness & emotional theory of mind   (Lane, et al., 2015^1,2^, Stonington, et al., 2013^1,2,6^)   1. Acceptance and clarity of emotions (Del Rio-Casanova, et al., 2018^4^) 2. Engaging in goal directed behaviour (Del Rio-Casanova, et al., 2018^5^) 3. Control of emotional impulses (Del Rio-Casanova, et al., 2018^7^) 4. Attending to emotions (Del Rio-Casanova, et al., 2018^6^) | 1. Level of Emotional Awareness Scale 2. Reading the Mind in the Eyes Test 3. Emotional Content in Frith Happe Animations Task 4. DERS- Clarity and Accept 5. DERS-Goals 6. DERS-Awareness 7. DERS-Lack of Emotional Control Scale |
| **Functional motor disorders** | 1. Emotional theory of mind   (Demartini, et al., 2014^2^) |  |
