# Supplementary material for: Emotion regulation in patients with somatic symptom and related disorders: A systematic review
Source: PLoS One. 2019 Jun 7;14(6):e0217277. doi: 10.1371/journal.pone.0217277 (PMC6555516; doi:10.1371/journal.pone.0217277)
Supplement: S4 Table — (DOCX) [file pone.0217277.s007.docx]

**S4 Table. Emotion Regulation Variables Examined in each Diagnostic Group**

| **ER Variables** | **Measures** | **Diagnostic groups (Reference^measure^)** |
| --- | --- | --- |
| **Attention** |  |  |
| **Attention switching** | 1. Experimental manipulation through thought suppression 2. Task switching paradigm 3. Emotional Stroop Test | 1. Chronic low-back pain (Burns, et al., 2011^1^) 2. Psychogenic non-epileptic seizures (Gul & Ahmad, 2014^2^) 3. Psychosomatic disorders (Wingenfeld, et al., 2014^3^) |
| **Attending to emotions** | 1. Five Facet Mindfulness Questionnaire, Observe subscale 2. Mindful Attention Awareness Scale 3. Difficulties in Emotion Regulation Scale-Awareness Subscale 4. Emotion Regulation Skills Questionnaire, Awareness Subscale | 1. Conversion Disorders (Del Rio-Casanova, et al., 20183) 2. Fibromyalgia (Veehof, et al., 20111) 3. Functional gastrointestinal disorders (Mazaheri, 20152) 4. Medically unexplained pain (Chavooshi, et al., 2016) 5. Medically Unexplained Symptoms with and without Depression (Schwarz, et al., 2017) 6. Psychogenic non-epileptic seizures (Brown, et al., 20133; Uliaszek et al., 20123) |
| **Goal directedness when emotionally distressed** | Difficulties in Emotion Regulation Questionnaires-Goals subscale | 1. Conversion Disorders (Del Rio-Casanova, et al., 20181) 2. Functional gastrointestinal disorders (Mazaheri, 20151) 3. Psychogenic non-epileptic seizures (Brown, et al., 20131; Uliaszek, Prensky & Baslet, 20121) |
| **Anger expression and Anger suppression** | 1. Anger Expression Inventory 2. Electronic Diary 3. State Trait Anger Expression Inventory 4. Self-Expression and Control Scale 5. Experimental manipulation of anger expression | 1. Chronic low back pain (Bruehl, et al., 2007^1^; 2012^1^; Burns, et al., 2008^1,5^; 2011^1^, Burns & Gerhart, et al., 2015^2^; 2016^2^ 2. Fibromyalgia (Sayar, et al., 2004^3^; van Middendorp, 2008^4^; 2010^4^) 3. Irritable bowel syndrome (Zoccali, et al., 2006^3^) 4. Myofascial pain (Castelli, et al., 2013^3^) |
| **Autonomic nervous system activity** | 1. Heart Rate Variability 2. Heart Rate 3. Skin Conductance Response 4. Respiration Rate 5. Affect modulated Startle in the Eye-Blink 6. Manipulation of Muscle Relaxation 7. Electromyogram 8. Respirators Sinus Arrhythmia) 9. Cortisol levels | 1. Chronic whiplash associated disorders (Koenig, et al., 2015^1^) 2. Functional abdominal pain (Walker, et al., 2017) 3. Interstitial cystitis/painful bladder syndrome (Twiss, et al., 2009^5^) 4. Irritable bowel syndrome (Elsenbruch, et al., 2010^6^, Fournier, et al., 2018^1,2,9^) 5. Multisomatoform disorders (Pollatos, Dietel, et al., 2011^1,2,3,4^; Pollatos, Herbert at al., 2011^1,2,3^) 6. Persistent somatoform pain disorders (Kleiman, et al., 2016^7,2,3^) 7. Psychogenic movement disorder (Seignourel, et al., 2007^5^) 8. Psychogenic non-epileptic seizures (Roberts, et al., 2012^2,8^) |
| **Expressive suppression & Emotional expression** | 1. Emotion Regulation Questionnaire 2. The Specific Affect Coding System 3. Emotional Facial Action Coding System 4. Emotional Approach Coding Scale 5. Cortauld Emotional Control Scale 6. The Affect Consciousness Interview 7. Observational coding | 1. Chronic pain (Chavooshi, et al., 2016^1^; Leong, et al., 2011^2^; Merten & Brunnhuber, 2004^3^; Wong & Fielding, 2013^1^) 2. Fibromyalgia (Geenen, et al., 2012^4^; van Middendorp, 2008^1,4^) 3. Functional neurological symptoms (Steffen, et al., 2015^1^) 4. Psychogenic non-epileptic seizures (Gul & Ahmad, 2014^1^; Urbanek, et al., 2014^5^, Roberts, et al., 2012^7^) 5. Somatoform Disorders (Waller & Scheidt, 2004^6^) 6. Psychosomatic Disorders (Rasting et al., 2005^3^) 7. Irritable bowel syndrome (Fournier at al., 2018^3^) |
| **Impulse control difficulties** | Difficulties in Emotion Regulation Questionnaire, Impulse Subscale | 1. Conversion disorders (Rio-Casanova, et al., 2018^1^) 2. Functional gastrointestinal disorders (Mazaheri, 2015^1^) 3. Psychogenic non-epileptic seizures (Brown, et al., 2013^1^; Uliaszek et al., 2012^1^) |
| **Emotional decision making based on bodily signals/ Perception of bodily signals** | 1. Iowa Gambling Task 2. Emotion Regulation Skills Questionnaire, Sensations Subscale | 1. Fibromyalgia (Walteros, et al., 2011^1^) 2. Medically unexplained symptoms (Schwarz, et al., 2017^2^) |
| **Emotional awareness, Emotional theory of mind**  **Emotion recognition** | 1. Facially Expressed Emotion Labeling 2. Level of Emotional Awareness Scale 3. Reading the Mind in the Eyes Test 4. Empathy Quatient 5. Ekman 60 6. Emotional Content in Frith Happe Animations Task 7. Karolinska Directed Emotional Faces battery 8. Comprehensive Affect Testing System 9. Tübinger Affekt Batterie 10. Affect Consciousness Interview 11. Ekman & Friesen faces 12. Animated morphing paradigm 13. Modified Affect Labeling Task 14. Emotion Regulation Skills Questionnaire, Understanding Subscale | 1. Chronic facial pain (Piekarzt, et al., 2015^1^) 2. Chronic muskuloskeletal pain (Burger, et al., 2016^2^) 3. Conversion disorder (Lane, et al., 2013^2,3^; Stonington, et al., 2013^2,3,6^) 4. Fibromyalgia (Di Tella, et al., 2015^3,4,5^) 5. Functional motor disorders (Demartini, et al., 2014^3^) 6. Functional somatic syndrome (Lane, et al., 2013^2,3^; Stonington, et al., 2013^2,3,6^) 7. Irritable bowel syndrome (Constantinou, et al., 2014^13^) 8. Medically unexplained symptoms (Schwarz, et al., 2017^14^) 9. Multisomatoform disorders (Pollatos, Herbert, et al., 2011^7^) 10. Somatoform disorders (Beck, et al., 2013^8^; De-Greck, et al., 2011^9^; Pedrosa Gil, et al., 2008^1^; Subic-Wrana, et al., 2010^2,6^; Waller & Scheidt, 2004^2,10^) 11. Somatic symptom disorders (Ozturk, et al., 2016^11^) 12. Psychogenic non-epileptic seizures (Schonenberg, et al., 2015^12^) 13. Temporamandibular disorders (Hass, et al., 2013^1^) |
| **Beliefs about and attitude to emotions** | 1. Beliefs about Emotions Scale 2. Difficulties in Emotion Regulation Scale (Clarity, Strategies and Accept Subscales) 3. Five Facet Mindfulness Questionnaire (Non-judge & describe subscales) 4. Trait Meta-Mood Scale 5. Acceptance and Action Questionnaire-II 6. Emotion Regulation Skills Questionnaire, (Clarity, Acceptance, Tolerance Subscales) 7. Cognitive Emotion Regulation Questionnaire, Acceptance subscale 8. Affective Style Questionnaire (adjust, tolerate, conceal subscales) | 1. Chronic fatigue syndrome (Rimes & Chalder, 20101) 2. Conversion Disorders (Del Rio-Casanova, 20182) 3. Fibromyalgia (Veehof, et al., 20113; Zautra, et al., 20014) 4. Functional dyspepsia, (Mazaheri, et al., 20167) 5. Functional gastrointestinal disorders (Mazaheri, 20152) 6. Medically unexplained symptoms (Schwarz, et al., 20176) 7. Psychogenic non- epileptic seizures (Brown, et al., 20132; Uliaszek, Prensky, & Baslet, 20122; Urbanek, et al., 20141, Baslet, et al., 20175,8) |
| **Reappraisal, automatic thoughts, efficacy in emotion regulation** | 1. Assessing Emotions Scale (Efficacy, Appraisal and Utilization Subscales) 2. Emotion Regulation Questionnaire 3. Automatic Thoughts Scale 4. Affective Memory Performance Test 5. Emotion Regulation Skills Questionnaire, (Modification and Self-support Subscales) 6. Cognitive Emotion Regulation Questionnaire | 1. Chronic pain (Agar-Wilson & Jackson, 2012^1^; Wong & Fielding, 2013^2^) 2. Fibromyalgia (Geenen et al., 2012^2^; van Middendorp, et al., 2008^2)^ 3. Functional dyspepsia (Mazaheri, et al., 2016^6^) 4. Functional neurological symptoms (Kienle, et al., 2018^2^) 5. Irritable bowel syndrome (Kilkens, et al., 2004^4^) 6. Medically unexplained pain (Chavooshi, et al., 2016^2^) 7. Medically unexplained symptoms (Schwarz, et al., 2017^5^) 8. Psychogenic non-epileptic seizures (Gul & Ahmad; 2014^2^) 9. Tension-type headache (Yucel, et al., 2002^3^) |
