# Supplementary material for: Emotion regulation in patients with somatic symptom and related disorders: A systematic review
Source: PLoS One. 2019 Jun 7;14(6):e0217277. doi: 10.1371/journal.pone.0217277 (PMC6555516; doi:10.1371/journal.pone.0217277)
Supplement: S5 Table — (DOCX) [file pone.0217277.s008.docx]

S5 Table. Study Characteristics and Summaries of Articles that examined Emotion Regulation involving mainly Attentional Processes

| **Diagnostic details & control condition (number of participants)** | **Authors** | **Emotion regulation measures** | **Psychosomatic symptom variables (Measure)** | **Design** | **Quality assessment^1^** | **Results** |
| --- | --- | --- | --- | --- | --- | --- |
| **Attention switching** | | | | | | |
| Psychogenic non-epileptic seizures (72)  & healthy controls (72) | Gul & Ahmad  (2014)[78] | Task-  switching paradigm | Psychological  distress (DASS) | E | +++ | Patients showed more difficulty in switching their attention from the emotional dimension of pictures than from the age dimension, indicating more cognitive costs for emotional features of pictures. Controls, on the other hand, showed equal switching performance for the emotion and age dimensions of the pictures. This attentional bias towards emotional dimensions was related to higher emotion suppression and lower cognitive reappraisal. |
| Psychosomatic  disorders (82) & healthy controls (39) | Wingenfeld, et al. (2011)[79] | Emotional Stroop test | Adverse childhood  experiences (CTQ),  anxiety and  depression (HADS), bodily symptoms  (FBL), dissociative  experiences (DES) | E | ++ | Emotion suppression, somatic symptoms, depression, childhood trauma, and dissociation were the significant predictors of mean reaction times in disengaging attention from the affective content of the words. |
| Chronic low back pain (58) | Burns et al. (2011)[80] | Manipula­tion of thought suppression during anger induction | Current pain behavior  (structured pain  behavior task) | E | ++ | Through an ironic effect of attention, anger report was higher in the suppression group compared to the no-suppression group. Suppression of emotional thoughts was positively related to pain behaviors in patients with higher trait anger-out and negatively related to pain behaviors in patients with higher trait anger-in. |
| Chronic fatigue syndrome (80) and healthy controls (80) | Rimes, Ashcroft, Bryan, & Chalder, (2016)[81] | Manipulation of thought suppression during a distressing film. | State affect and fatigue (VAS) | E | ++ | Through an ironic effect of attention, in the suppression condition, regardless of the group, anxiety levels increased from pre to post film-watching. Suppression condition did not affect self-reported fatigue of the patients. |
| **Goal directedness when emotionally distressed** | | | | | | |
| Functional  gastrointestinal  disorders (167) | Mazaheri  (2015)[85] | Difficulties in Emotion Regulation Scale, GOALS Subscale | Depression, anxiety and stress (DASS),  gastrointestinal  symptoms (GSRS) | CS | +++ | Difficulties in engaging attention to one’s current task when emotionally distressed predicted gastrointestinal symptoms in the patients. |
| Conversion disorders (43) & healthy controls (42) | Del Rio-Casanova et al. (2018) [84] | Difficulties in Emotion Regulation Scale, GOALS Subscale | Depression, anxiety (HADS), somatoform dissociation (SDQ-20), psychoform dissociation (DES-II) | CC | +++ | The patient groups reported greater difficulties in focusing on their tasks when they are distressed, compared to healthy controls. After stepwise elimination of DERS factors, this factor remained a significant predictor of patient status. |
| Psychogenic non-  epileptic seizures  (PNES) (43) &  epilepsy (24) | Brown et al. (2013)[25] | Difficulties in Emotion Regulation Scale, GOALS Subscale | Anxiety (GAD-7), depression (PHQ-9), somatization (SDQ-20), attachment styles  (RSQ) | CC | +++ | Patients with PNES reported greater difficulties in focusing on their tasks when they are distressed. This difference was more pronounced between the emotionally dysregulated PNES group and the epilepsy patients. |
| Psychogenic non-  epileptic seizures  (70) | Uliaszek et al. (2012) [83] | Difficulties in Emotion Regulation Scale, GOALS Subscale | Depression (BDI-II), dissociative  experiences (DES),  psychological distress (DASS), functioning  and physical distress  (PHQ-15, DFI) | CS | ++ | Patients with PNES reported greater difficulties in focusing on their tasks when they are distressed compared to normative data, which was more obvious in the emotionally dysregulated patient group. |
| **Attending to emotions** | | | | | | |
| Fibromyalgia (141) | Veehof et al. (2011) [86] | Five Facet Mindfulness Question­naire, Observe Subscale | Neuroticism and  openness to new  experiences (NEO-FFI), anxiety and  depression (HADS),  mental and physical  health (SF-12) | CS | + | The capacity to observe, notice, and attend to subjective experience was negatively related to alexithymia and positively related to openness to experience. Acting with awareness, such as doing things by paying attention, was positively correlated with acceptance of experiences and negatively correlated with alexithymia, neuroticism, depression, and anxiety. |
| Functional  gastrointestinal  disorders (167) | Mazaheri  (2015) [85] | 1. Mindful Attention Awareness Scale.  2. Difficulties in Emotion Regulation Scale, AWARE­NESS Subscale | Depression, anxiety  and stress (DASS),  gastrointestinal  symptoms (GSRS) | CS | +++ | Mindful attention negatively predicted depression, anxiety, and stress. Mindful attention was also negatively correlated to all facets of emotion dysregulation. |
| Medically unexplained  pain (100) | \| Chavooshi et al. (2016) [87] \| \| --- \| | Mindful Attention Awareness Scale | Pain intensity  (NPRS), depression  & anxiety (DASS-21), quality of life (QOLI) | I | ++ | Patients treated with intensive, short-term dynamic psychotherapy had improved mindful attention following the psychotherapy, compared to patients who received treatment as usual. Other psychosomatic variables were also much improved compared to the control group. |
| Medically unexplained symptoms (MUS) (138), MUS comorbid with major depressive disorder (MDD) (114), MDD (106), healthy controls (100) | Schwarz et al. (2017) [116] | Emotion Regulation Skills Question­naire, Awareness Subscale | Physical complaints (SOMS-7T), depression (BDI-II), symptom checklist (SCL-90) | CC | ++ | No difference was found in the capacity for attending to emotions between MUS patients and healthy controls, or between MUS and MDD patients or MUS and MUS+MDD patients. Only between MUS+MDD and healthy controls was the awareness score different, being higher in the control group. |
| Conversion disorders (43) & healthy controls (42) | Del Rio-Casanova, et al. (2018) [84] | Difficulties in Emotion Regulation Scale, AWARE­NESS Subscale | Depression, anxiety (HADS), somatoform dissociation (SDQ-20), psychoform dissociation (DES-II) | CC | +++ | The capacity of attending to and acknowledging emotions was decreased in the patient group, compared to controls. |
| Psychogenic non-epileptic seizures  (PNES) (43) &  epilepsy (24) | Brown et al. (2013) [25] | Difficulties in Emotion Regulation Scale, AWARE­NESS Subscale | Anxiety  (GAD-7),  depression (PHQ-9), somatization (SDQ-20), attachment styles (RSQ) | CC | +++ | Capacity to attend to emotions and acknowledge them did not differ between the two patient groups, or between clusters based on emotion dysregulation level. Authors propose a possible reliability or validity problem with the AWARENESS Subscale. |
| Psychogenic non-  epileptic seizure  (70) | Uliaszek et al. (2012) [83] | Difficulties in Emotion Regulation Scale, AWARE­NESS Subscale | Depression (BDI-II), dissociative  experiences (DES),  psychological distress (DASS), functioning and physical distress  (PHQ-15, DFI) | CS | ++ | The capacity for attending to and acknowledging emotions was not different from the normative sample and slightly higher in the emotionally dysregulated patient group, compared to the patient group with elevated emotion regulation. |

^1^Quality of the studies was rated with +, ++, or +++ when 25–49%, 50–79%, or 80% or more of the criteria were rated with “yes”.

**Abbreviations of the study designs**

**CS:** Cross Sectional  **CC:**Case Control **E:**Experimental **L:**Longitudinal **I:**Intervention/psychotherapy study

**Abbreviations of the symptom measures & paradigms**

**BDI:** Beck Depression Inventory, **CTQ:** Childhood Trauma Questionnaire, **DASS:** Depression, Anxiety and Stress Symptoms , **DES:** Dissociative Experiences Scale, **DFI:** Disruption of Functioning Index, **FBL:** Freiburger Beschwerdeliste-Revised, **GSRS:** Gastrointestinal Symptom Rating Scale, **GAD:** Generalized Anxiety Disorder Questionnaire, **HADS:** Hospital Anxiety and Depression Scale, **NEO-FFI:** NEO Five-Factor Personality Inventory, **NPRS:** Numerical Pain Rating Scale, **PHQ:** The Patient Health Questionnaire, **RSQ:** Responses to Stress Questionnaire, **QOLI:** Quality of Life Inventory, **SCL-90:** Symptom Checklist-90, **SDQ:** Somatoform Dissociation Questionnaire, **SF:** Short form Health Survey, **SOMS:** Screening for Somatoform Disorders
