# Supplementary material for: Emotion regulation in patients with somatic symptom and related disorders: A systematic review
Source: PLoS One. 2019 Jun 7;14(6):e0217277. doi: 10.1371/journal.pone.0217277 (PMC6555516; doi:10.1371/journal.pone.0217277)
Supplement: S6 Table — (DOCX) [file pone.0217277.s009.docx]

S6 Table. Study Characteristics and Summaries of Articles that examined Emotion Regulation involving mainly Bodily Processes

| **Diagnostic details & control condition (number of participants)** | **Authors** | **Emotion regulation measures** | **Psychosomatic symptom variables (Measure)** | **Design** | **Quality assessment^1^** | **Results** |
| --- | --- | --- | --- | --- | --- | --- |
| **Anger expression & anger suppression** | | | | | | |
| Chronic low back pain (13) & healthy controls  (14) | Bruehl et al.  (2007) [89] | Anger Expression Inventory | Ongoing and general pain (pain manipulation & MPQ-short), blood  pressure, plasma beta-  endorphin level, anxiety | E | ++ | Greater trait anger-out and anger-in were associated with greater acute pain in patients. Pain-induced beta-endorphin release was related to lower pain in both groups. Greater anger-out scores predicted smaller beta-endorphin release. |
| Chronic low back  pain (84) | Burns et al. (2008) [97] | 1. Anger Expression Inventory  2. Manipulation of expression vs. inhibition during experimental harassment paradigm | Symptom-specific muscle  reactivity (EMG) | E | ++ | High trait anger-out patients in the anger inhibition condition showed the greatest lower paraspinal reactivity followed by the slowest recovery. High trait anger-out in anger expression condition showed the highest SBP followed by rapid recovery. Higher trait anger-out scores were related to higher heart rate reactivity during experimental harassment. |
| Chronic low back  pain (58) | Burns et al. (2011) [80] | Anger Expression Inventory | (STAI), depression (BDI),  current pain behavior  (structured pain behavior  task) | E | ++ | Trait anger expression style interacted with the experimental thought suppression on predicting pain behavior. Trait anger-out was related to more pain behavior during the suppression condition, but less pain behavior during the no-suppression condition. |
| Chronic low back  pain (48) &  healthy controls  (36) | Bruehl et al. (2012) [90] | 1. Anger Expression Inventory  2. Electronic Diary | Pain (electronic diary),  depression (BDI), anxiety  (STAI) | E | +++ | Patients showed higher daily anger expression. Greater state anger expression affected subsequent time-lagged pain intensity. Trait anger expression moderated the relationship between state anger expression and chronic pain. |
| Chronic low back pain of patients (105) & their spouses (36) | Burns et al. (2015; 2016)^3^[92,91] | Electronic diary from both patients and their spouses | 1.Perceived criticism, hostility, and support by both patients and spouses  2. Electronic diary for state pain and pain interference | L (14 days) | ++ | As patients’ anger arousal increased, perceived criticism, hostility, and negative affect of the spouse also increased, especially in couples with a male patient. Only in the male patients was anger expression associated with perceived criticism, hostility, and negative affect in the spouses. The predominant relationship between anger expression and response of the spouse was rather concurrent than lagged. Greater anger inhibition was related to decreased concurrent spouse criticism. Anger expression and inhibition were also related to patient- and spouse-reported pain and pain interference. |
| Myofascial pain  (45) & healthy  controls (45) | Castelli, et al. (2013) [93] | State Trait Anger Expression Inventory-II | Medical evaluation,  psychological distress  (BDI, STAI, Distress  Thermometer) | E | ++ | Myofascial pain patents showed a greater tendency to suppress anger expression (anger-in). Anger-in was correlated with depression, anxiety, and alexithymia in patients. |
| Fibromyalgia  (FA) (50),  rheumatoid  arthritis (20) &  healthy controls  (42) | Sayar et al. (2004) [94] | State Trait Anger Expression Inventory | Pain (VAS), fibromyalgia  impact (FIQ), depression  (BDI), anxiety (BAI) | CC | +++ | Anger-in was greater in fibromyalgia patients than in rheumatoid arthritis patients and healthy controls. Behavioral expression of anger, together with anxiety, predicted the severity of the pain. |
| Fibromyalgia  (403) & healthy  controls (196) | van Midden­dorp  et al. (2008) [96] | Self-Expression and Control Scale | Pain (MPI), fibromyalgia  impact (FIQ), mental distress (MPI & FIQ) | CC | ++ | Internalization of anger was positively correlated with mental distress. |
| Fibromyalgia  (333) | van Midden­dorp et al. (2010) [95] | 1. Self-Expression and Control Scale  2. Diary | Pain (Diary) | CS | +++ | Trait anger inhibition, but not state anger inhibition, was related to end-of-day-pain. The lowest pain was reported in patients with high trait anger expression who actually expressed their anger (state anger expression). |
| Irritable bowel  syndrome (52) & healthy volunteers (100) | Zoccali et al. (2006) [88] | State Trait Anger Expression Inventory | Defense mechanisms  (DMI) | CC | ++ | No statistical difference was found between groups in expression or suppression of anger. |
| **Expressive suppression and emotional expression** | | | | | | |
| Chronic pain (224) | Wong & Fielding (2013) [101] | Emotion Regulation Questionnaire | Pain (CPQ), pain catastro­phizing (PCS) |  | ++ | Expressive suppression was related to pain catastrophizing and mediated the relationship between negative affect and pain catastrophizing. |
| Chronic pain  (100) (in the  article cited as  medically  unexplained pain) | Chavooshi et al. (2016) [87] | Emotion Regulation Questionnaire | Pain intensity (NPRS),  depression & anxiety (DASS-21), quality of life (QOLI) | I | ++ | Patients treated with intensive short-term dynamic psychotherapy reported significantly reduced suppression compared to patients who received treatment as usual, along with other decreasing psychosomatic complaints. |
| Somatic symptom disorders (35) & healthy controls (35) | Erkic, Bailer, Fenske, et al., (2017) [100] | Emotion Regulation Questionnaire | Number and intensity of symptoms (SOMS), symptom severity (PHQ-15), life disruption (PDI), depression (BDI) | E | +++ | A trend was found towards more expressive suppression in the patients compared to controls. |
| Fibromyalgia (403) | Geenen et al. (2012) [102] | Emotional Approach Coping Scale | Fibromyalgia impact (FIQ) | CS | ++ | Patients who experience their emotions intensely but suppress their emotional expressions suffer more from the impact of fibromyalgia. |
| Fibromyalgia  (403) & healthy  controls (196) | van Midden­dorp et al. (2008) [96] | Emotional Approach Coping Scale,  Emotion Regulation Questionnaire | Pain (MPI), fibromyalgia  impact (FIQ), mental distress (MPI & FIQ) | CC | ++ | Patients’ general emotional expression was lower than that of the controls, and was negatively correlated with negative affect and mental distress. Expressive suppression was positively correlated with negative affect and mental distress. |
| Functional  neurological  symptoms (45) & healthy controls  (45) | Steffen et al. (2015) [98] | Emotion Regulation Questionnaire | Functional neurological  symptoms (SDQ), early  trauma (ETI), stressful life  events (LEQ) | CC | ++ | Patients reported greater suppressive emotion regulation, which was positively associated with the severity of the symptoms. |
| Psychogenic non-epileptic seizures (72) & healthy controls (72) | Gul & Ahmad  (2014) [78] | Emotion Regulation Questionnaire | Psychological distress  (DASS) | E | +++ | Patients scored higher on expressive suppression. Greater expressive suppression was related to poorer cognitive flexibility. |
| Psychogenic non-epileptic seizures (56) & healthy controls (88) | Urbanek et al. (2014) [99] | Courtauld Emotional Control Scale | Depression (HADS),  seizure characteristics | CC | ++ | Patients reported greater control over their emotional reactions than controls, especially for anxiety and sadness behavior. |
| Psychogenic non-epileptic seizures (PNES) (18), seizure-free individuals with high (18) and low (18) posttraumatic stress symptoms (PTS) | Roberts et al. (2012) [108] | Emotional behavior in response to positive, negative, and neutral affective pictures | Trauma (PCL-S), psychiatric distress (SCL-90R), | E | + | Fewer PNES patients than PTS-high participants displayed positive emotional behavior. PNES and PTS-low patients did not differ. PNES patients did not differ from PTS-high or -low individuals in negative emotional behavior. |
| Somatoform  disorders (40) &  healthy controls  (20) | Waller & Scheidt (2004) [103] | The Affect Consciousness Interview | Somatoform symptoms  (SOMS), negative affect  (HADS) | CC | +++ | Patients reported lower capacity to nonverbally express their feelings than controls. Nonverbal expressiveness was negatively correlated with the dimensions of alexithymia. |
| Irritable bowel syndrome (52) | Sibelli, Chalder, Everitt, Workman, Bishop & Moss-Morris (2017) [104] | Semi-structrured interviews |  | CS, qualitative | n.a (qualitative) | When experiencing negative emotions, patients spoke about bottling up emotions but not expressing them, for reasons such as believing that expressing does not make a change, fear of rejection or not wanting to worry others. |
| Chronic fatigue syndrome (80) and healthy controls (80) | Rimes, Ashcroft, Bryan, & Chalder, (2016) [81] | Observer ratings of emotional expression in suppression and expression choice conditions (FACES), Self-ratings of emotional suppression and expression (VAS) | Anxiety and depression (HADS) | E | ++ | Patients expressed lower number and intensity of emotions than healthy controls in both expression and suppression conditions. This effects remained significant after controlling for anxiety and depression. |
| Chronic pain (21), healthy controls (11) & therapist | Merten &  Brunnhuber  (2004) [106] | Emotional Facial Action Coding System during interviews | Psycho­dynamic character  of the patients (OPD) | E | + | The total negative expressions (anger, disgust, contempt) were higher in patients. Patients expressed more frequent contempt and controls expressed more genuine joy. Therapists’ facial expressions involved more negative emotions when talking to patients. Negative expressions were negatively correlated to level of personality organization. The congruence between negative emotional experience and expression was found only in patients. |
| Psychosomatic  disorders (12) | Rasting et al. (2005) [105] | Emotional Facial Action Coding System during interviews | - | E | + | Patients’ expressivity of aggressive affect, especially contempt, was negatively related to their alexithymia level. Therapists’ facial response was distinguished by expression of contempt. |
| Irritable bowel syndrome (25) & healthy controls (26) | Fournier et al. (2018) [109] | Emotional Facial Action Coding System while watching frightening film | Anxiety (STAI-Y), depression (CES-D) | E | ++ | Patients displayed more sadness and tended to display more rage than healthy controls. |
| Chronic pain (78) (patients and their spouses) | Leong et al. (2011) [107] | The Specific Affect Coding System during couple interviews | Pain (MPI), psychological  distress (MASQ), dyadic  adjustment (DAS) | E | ++ | Significant interaction was found between patient gender and sequences of invalidation and validation. Only male patient couples’ reciprocal invalidation was related to worse pain. Spouses’ indiscriminate validation was associated with poorer pain and relationship satisfaction in couples in which the patient was male. |
| **Autonomic nervous system activity** | | | | | | |
| Chronic whiplash-  associated  disorders (30) &  healthy controls  (31) | Koenig et al.  (2015) [110] | Vagally mediated  heart rate variability | Pain catastrophizing (PCS) | CC | + | Patients presented lower vagally mediated HRV  (high frequency-HRV), indicating a lower parasympathetic activation during resting state.  They also reported higher pain catastrophizing,  which was inversely correlated with vagally mediated HRV |
| Multisomatoform  disorder (21) &  healthy controls  (21) | Pollatos, Dietel,  et al. ^2^ (2011) [71] | Heart rate, heart rate variability, skin conductance response, & respiration rate | Pain threshold and pain  tolerance (pain  manipulation and pain  imagination), depression  (BDI) | E | +++ | Patients showed lower parasympathetic and higher sympathetic activation during baseline, experimentally induced pain, and pain imagination. Patients had decreased pain tolerance. Controls showed greater vagal withdrawal during pain assessment, which correlated with increased pain tolerance. |
| Multisomatoform disorder (23) & healthy controls (23) | Pollatos, Herbert,  et al. ^2^(2011) [111] | Heart rate, heart rate variability, skin conductance response | Depression (BDI),  somatosensory  amplification (SAS), trait  anxiety (STAI) | E | * | Patients showed lower parasympathetic reactivity during emotion recognition and emotion appraisal tasks, and increased sympathetic activation during baseline. |
| Persistent  somatoform pain disorder (42) | Kleiman et al. (2016) [112] | Electromyo­gram, heart rate, skin conductance response |  | E | +++ | During a stressful task (oral presentation), high alexithymic patients showed significantly lower skin conductance and reported greater negative affect than low alexithymic patients. Alexithymic patients did not have increased autonomic arousal at baseline, relaxation, or stress compared to non-alexithymic patients. |
| Chronic fatigue syndrome (80) and healthy controls (80) | Rimes, Ashcroft, Bryan, & Chalder, (2016) [81] | Skin conductance response | Fatigue (VAS) | E |  | Patients showed higher skin conductance during stressful film watching task, but not during baseline, compared to controls. This effect remained significant after controlling for anxiety and depression. Increases in fatigue was positively related to skin conductance response during baseline and film watching only in the patient group. |
| Psychogenic  movement  disorder (12) &  healthy controls  (12) | Seignourel et al. (2007) [114] | Affect-modulated startle in  eye blink (EMG) | Depression (BDI), trait  anxiety (STAI) | E | +++ | Patients showed higher startle responses to both positive and negative pictures than neutral ones, as compared to control subjects. Controls showed highest startle responses to negative pictures, followed by positive and neutral ones. |
| Psychogenic non-epileptic seizures (18), seizure-free individuals with high (18) and low (18) posttraumatic stress symptoms | Roberts et al. (2012) [108] | Heart rate & respiratory sinus arrhythmia (RSA) | Trauma (PCL-S), psychiatric distress (SCL-90R), | E | + | PNES patients did not differ from seizure-free PTS-low or -high individuals in their average HR and RSA while looking at pictures. However, during a resting state, patients showed lower RSA than PTS-low, but did not differ from PTS-high. |
| Interstitial  cystitis/painful  bladder syndrome  (13) & healthy  controls (16) | Twiss et al.  (2009) [113] | Affect-modulated startle in  eye blink (EMG) | Pain (VAS during visceral  threat), anxiety and  depression (HADS) | E | ++ | Patients had greater acoustic startle reflex than controls in non-imminent threat conditions (baseline, safe, and anticipation phases). Patients and controls showed similar robust responses in imminent threat conditions. |
| Irritable bowel syndrome (25) & healthy controls (26) | Fournier et al. (2018) [109] | Heart rate, heart rate variability, cortisol levels | State anxiety (STAI-Y), depression (CES-D). | E | ++ | Patients showed a parasympathetic withdrawal from baseline to fear-eliciting film watching, whereas healthy controls did not. Patients also showed an increase in their heart rate from baseline to fearful task, while HC did not. There was no correlation between patients’ expressiveness and their physiological responses. |
| Irritable bowel  syndrome (IBS)  (15) & healthy  controls (12) | Elsenbruch, et al.  (2010) [115] | Progressive muscle  relaxation (experi­mental  manipula­tion) | Pain (pain manipulation),  anxiety and depression  (HADS), anxiety (STAI) | E | ++ | In both stress and relaxation conditions, patients reported higher pain ratings for painful and non- painful rectal distensions than controls. |
| **Impulse control difficulties, action tendency, action readiness** | | | | | | |
| Psychogenic non-epileptic seizure  (70) | Uliaszek et al. (2012) [83] | Difficulties in Emotion Regulation Scale, Impulse Subscale | Depression (BDI-II),  dissociative experiences  (DES), psychological  distress (DASS),  functioning and physical  distress (PHQ-15, DFI) | CS | ++ | From two empirically established clusters, one had significantly increased impulse control difficulties as compared to normative data. This cluster was also associated with higher rates of comorbid psychiatric symptoms and life impairment. The other cluster’s impulsivity score did not differ from the normative data. |
| Psychogenic non-epileptic seizures  (PNES) (43) &  epilepsy (24) | Brown et al.  (2013) [25] | Difficulties in Emotion Regulation Scale, Impulse Subscale | Anxiety (GAD-7), depression (PHQ-9),  somatization (SDQ-20),  attachment styles (RelSQ) | CC | +++ | Patients with PNES reported greater impulse control difficulties compared to epilepsy patients. This difficulty was more pronounced in the emotionally dysregulated cluster of PNES patients, who also had higher alexithymia, somatization, and psychopathology scores. |
| Conversion disorders (43) & healthy controls (42) | Del Rio-Casanova et al. (2018) [84] | Difficulties in Emotion Regulation Scale (DERS), Lack of Emotional Control Subscale | Depression, anxiety (HADS), somatoform dissociation (SDQ-20), psychoform dissociation (DES-II) | CC | +++ | Patients reported greater difficulties in emotional action control compared to controls. After stepwise elimination of DERS factors, this factor remained a significant predictor of patient status. |
| Functional  gastrointestinal  disorders (167) | Mazaheri (2015) [85] | Difficulties in Emotion Regulation Scale, Impulse Subscale | Depression, anxiety and  stress (DASS),  gastrointestinal symptoms  (GSRS) | CS | +++ | Patients’ difficulty in controlling their behavior when they are emotionally distressed independently predicted their gastrointestinal symptoms and anxiety. |
| Medically unexplained symptoms (MUS) (138), MUS comorbid with major depressive disorder (MDD) (114), MDD (106), healthy controls (100) | Schwarz et al. (2017) [116] | Emotion Regulation Skills Question­naire, (Readiness to confront ) | Physical complaints (SOMS-7T), depression (BDI-II), symptom checklist (SCL-90) | CC | ++ | MUS patients were better than MUS+MDD patients in readiness to confront negative emotions. Only MDD+MUS patients scored lower than healthy controls in readiness to confront emotions, but not MUS or MDD patients. |
| **Emotional decision making based on bodily signals, perception of bodily signals** | | | | | | |
| Fibromyalgia  patients (15) &  healthy controls  (15) | Walteros et al. (2011) [117] | Iowa Gambling Task | Anxiety (STAI),  depression (BDI), general  cognitive functioning  (WAIS), cognitive  flexibility (Stroop test),  conditional associative  learning (CALT) | E | - | Patients selected more disadvantageous cards and showed more random behavior in the emotion-based decision task than did healthy controls. They also showed more perseveration errors in the CALT task. Groups did not differ in other standardized cognitive tests. The findings indicated a specific cognitive deficit regarding affective information rather than a general cognitive difficulty in the patients. |
| Medically unexplained symptoms (MUS) (138), MUS comorbid with major depressive disorder (MDD) (114), MDD (106), healthy controls (100) | Schwarz et al. (2017) [116] | Emotion Regulation Skills Question­naire, Sensations Subscale | Physical complaints (SOMS-7T), depression (BDI-II), symptom checklist (SCL-90) | CC | ++ | MUS patients were better at distinguishing bodily sensations than MUS+MDD patients. Healthy controls differed only from MUS+MDD patients, that ability being higher in the healthy control group. No difference was found between the capacity for perceiving bodily signals of emotions between MUS patients and healthy controls, or between MUS and MDD patients. |

^1^Quality of the studies was rated with +, ++, or +++ when 25–49%, 50–79%, or 80% or more of the criteria were rated with “yes.”

^2^ The two article findings are based on the same sample data.

^3^ The two article findings are based on the same sample data.

**Abbreviations of the study designs**

**CS:** Cross Sectional **CC:** Case Control **E:** Experimental **L:** Longitudinal **I:** Intervention/psychotherapy study

**Abbreviations of the symptom measures & paradigms**

**BDI:** Beck Depression Inventory, **CALT:** Conditional Associative Learning Task, **CES-D:** Center for Epidemiology Articles-Depression Scale, **CPQ:** Chronic Pain Questionnaire, **DAS:** The Dyadic Adjustment Scale, **DASS:** Depression, Anxiety and Stress Symptoms , **DES:** Dissociative Experiences Scale, **DFI:** Disruption of Functioning Index, **DMI:** Defense Mechanism Inventory, **ETI:** Early Trauma Inventory, **FACES:** Facial Expression Coding System, **FIQ:** The Fibromyalgia Impact Questionnaire, **GSRS:** Gastrointestinal Symptom Rating Scale, **GAD:** Generalized Anxiety Disorder Questionnaire, **HADS:** Hospital Anxiety and Depression Scale, **LEQ:** Life Events Questionnaire, **MASQ:** The Mood and Anxiety Symptom Questionnaire, **MPI:** Multidimensional Pain Inventory, **MPQ:** McGill Pain Questionnaire, **NPRS:** Numerical Pain Rating Scale, **OPD:** Operationalized Psychodiagnostic Manual, **PDI:** Pain Distruption Index, **PHQ:** The Patient Health Questionnaire, **RelSQ:** Relationship Scales Questionnaire, **QOLI:** Quality of Life Inventory, **SAS:** Somatosensory Amplification Scale, **SCL-90:** Symptom Checklist-90, **SDQ:** Somatoform Dissociation Questionnaire, **SOMS:** Screening for Somatoform Disorders, **STAI:** State Trait Anxiety Inventory, **WAIS:** Wechsler Adult Intelligence Scale, **VAS:** Visual Analog Scale
