# Supplementary material for: Emotion regulation in patients with somatic symptom and related disorders: A systematic review
Source: PLoS One. 2019 Jun 7;14(6):e0217277. doi: 10.1371/journal.pone.0217277 (PMC6555516; doi:10.1371/journal.pone.0217277)
Supplement: S7 Table — (DOCX) [file pone.0217277.s010.docx]

**S7 Table.** Study Characteristics and Summaries of Articles that examined Emotion Regulation involving higher-order Knowledge/Appraisal Processes

| **Diagnostic details &control condition (number of participants)** | **Authors** | **Measures** | **Psychosomatic symptom variables (Measure)** | **Design** | **Quality^1^** | **Results** |
| --- | --- | --- | --- | --- | --- | --- |
| **Emotional awareness, emotional theory of mind & emotion recognition** | | | | | | |
| Somatoform disorders (40) & healthy controls (20) | Waller & Scheidt (2004)[103] | 1. Level of Emotional Awareness Scale 2. Affect Consciousness Interview | Somatoform symptoms (SOMS), negative affectivity (HADS) | CC | +++ | A reduced awareness of emotions was found in patients based on ACI but not LEAS. Particularly awareness of anger, guilt, and mean negative affect was diminished in the patient group. Alexithymia was greater among patients. |
| Somatoform disorders (20) & healthy controls (20) | Pedrosa Gil et al. (2008) [128] | Facially Expressed Emotion Labeling | Somatoform symptoms (SOMS), psychological distress (SCL-90-R), depression (HAMD) | E | ++ | Patients recognized fewer emotional expressions than controls, which became non-significant when alexithymia was controlled. |
| Somatoform disorders (30) and healthy controls (30) | Subic-Wrana et al. (2010) [118] | 1. Level of Emotional Awareness Scale 2. Emotional Content in Frith-Happe-Animations Task | Theory of mind functioning/ mentalizing (FHAT), somatic and psychic symptoms (SCL-90- R) | E | ++ | Patients showed reduced emotional awareness in LEAS. Reduced emotional content and reduced emotional theory of mind functioning at FHAT was observed in patients. Theory of mind (FHAT) and emotional awareness (LEAS) together correctly classified 80% of the patients. |
| Somatoform disorders (20) & healthy controls (20) | De Greck et al. (2011) [126] | Tübinger Affekt Batterie | Somatoform symptoms (SOMS-2, BSI) | E | ++ | Somatoform patients showed more diminished mean emotion recognition and recognition of single emotions of anger, joy, sadness, and fear, but not of neutral emotions. |
| Somatoform disorders (35) & controls (73) | Beck et al. (2013) [125] | Comprehensive Affect Testing System | Somatoform symptoms (SOMS-2, BSI) | E | + | Patients demonstrated reduced abilities to recognize others’ affects. |
| Somatic symptom disorders (54) & healthy controls (46) | Ozturk et al. (2016) [127] | Ekman & Friesen Faces | Depression (BDI), anxiety (BAI) | E | +++ | Patients had lower scores in recognizing fear, disgust, and neutral faces. Mean alexithymia and its dimensions were also higher in patients than controls. After controlling for alexithymia, depression, and anxiety, the difference between patients and controls in emotion recognition diminished. |
| Somatic symptom disorders (35) & healthy controls (35) | Erkic, Bailer, Fenske, et al., (2017) [100] | NimStim set of facial expressions | Number and intensity of symptoms (SOMS), symptom severity (PHQ-15), life disruption (PDI), depression (BDI) | E | +++ | A trend was found in patients’ scores in recognition of more angry faces correctly. |
| Multisomatoform disorder (23) & matched healthy controls (23) | Pollatos, Herbert, et al. (2011) [111] | Karolinska Directed Emotional Faces battery | Depression (BDI), somatosensory amplification (SAS), trait anxiety (STAI) | E | +++ | Patients showed decreased emotion recognition for faces showing sad and neutral emotions even after alexithymia, depression, and anxiety were controlled for. |
| Medically unexplained symptoms (MUS) (138), MUS comorbid with major depressive disorder (MDD) (114), MDD (106), healthy controls (100) | Schwarz et al. (2017) [116] | Emotion Regulation Skills Questionnaire, (Understanding Subscale) | Physical complaints (SOMS-7T), depression (BDI-II), symptom checklist (SCL-90) | CC | ++ | MUS patients were better than MDD and MUS+MDD patients in their ability to understand and reflect on their own emotional experiences, but worse than healthy controls. |
| Chronic musculoskeletal pain (72) | Burger et al. (2016) [119] | Level of Emotional Awareness Scale | Pain intensity and interference (BPI), depression (CES-D), sensory and affective pain (MPQ), psychological distress (BSI), psychological attribution for pain | I (pre, post, & follow-up) | ++ | Patients’ emotional awareness, in particular for others’ emotions, increased from pre- to post- treatment (psychological attribution, emotional awareness and expression therapy). Accordingly, the alexithymia level significantly decreased. Patients’ psychological attribution for pain was also improved. Parallel with the findings, many of the pain and psychological symptom variables in the pre-treatment improved in the post-treatment and still increased slightly after 6-month follow-up. |
| Conversion disorder (29), functional somatic syndrome (FSS) (30) & explained medical disorder (30) | Stonnington et al. (2013)^2^ [124] | Emotional Content in Frith Happe Animations Task 2. Reading the Mind in the Eyes Test 3. Level of Emotional Awareness Scale | Cognitive theory of mind (MSS), depression, anxiety, & physical symptoms (MADRS, HAM-A, SCL-90), quality of life (SF-36) | E | +++ | Patients with conversion disorder and FSS scored lower than medical controls on the affective theory of mind task measured by FHAT-L, but not on EYES. They also reported greater anxiety and decreased positive affect. Patients with conversion disorder showed higher alexithymia than medical controls. |
| Conversion disorder (29), functional somatic syndrome (FSS) (30) & explained medical disorder (30) | Lane et al. (2015)^2^ [59] | 1. Level of Emotional Awareness Scale 2. Reading the Mind in the Eyes Test | Cognitive theory of mind (MSS), depression (MADR), quality of life (SF- 36), positive and negative affect (PANAS) | E | * | Emotional awareness measured with LEAS was positively related to affective and cognitive theory of mind after controlling for negative affect in the patients. |
| Functional motor disorders (55), organic movement disorders (33) & healthy controls (34) | Demartini et al. (2014) [122] | Reading the Mind in the Eyes Test | Depression (MADRS), personality disorders (SCID) | E | ++ | No difference was found between groups in the EYES test. |
| Psychogenic non- epileptic seizures (15) & healthy controls | Schönenberg et al. (2015) [131] | Animated morphing paradigm | Cognitive theory of mind (MASC), perceived stress (PSS), psychopathology (MINI) | E | ++ | Patients’ performance in the dynamic emotion recognition test was not different from the healthy controls. Divergence reported in the literature might have stemmed from methodological differences. |
| Fibromyalgia (40) & healthy controls (41) | Di Tella et al. (2015) [120] | 1. Reading the Mind in the Eyes Test 2. Empathy Quotient 3. Ekman 60 | Pain intensity (FIQ- Pain), anxiety and depression (HADS) neuropsychological assessment (short- term memory, learning, attention, executive functioning) | E | ++ | Patients had greater difficulty in attributing others’ affects compared to controls. Similarly, they showed reduced ability to recognize emotions, especially anger and disgust, and reported greater mean alexithymia, especially in identifying and describing feelings, compared to controls. In empathy, no difference was found between groups. The patients scored lower on many neuropsychological tests compared to controls; however, no significant correlation was found between neuropsychological variables and affective-social cognition variables. This suggests that affective-social cognition capacities are independent of the neuropsychological deficits. |
| Chronic facial pain | von Piekartz et al. (2015) [129] | Facially Expressed Emotion Labeling | Left/right facial movement judgment task, depression (BDI), pain intensity (CAS) | E | +++ | Patients were less accurate in total emotion recognition than controls. Accuracy in emotion recognition was negatively related to pain intensity. Patients were also less accurate and had longer response time in the left/right facial movement judgment task than controls, which indicated a disruption of body-part motor processing. The facial motor processing and facial emotion recognition were also related to each other, suggesting the emotion recognition problems not exclusively about emotion processing. |
| Temporomandibular disorders (20) & healthy controls (20) | Haas et al. (2013) [152] | Facially Expressed Emotion Labeling | Somatoform symptoms (SOMS- 2a), depression (HAMD), pain (German Pain Questionnaire) | E | +++ | Patients had more pronounced alexithymia and impaired total emotion recognition. Alexithymia together with somatization explained a higher proportion of variance of the emotion recognition scores, as compared to group membership. |
| Irritable bowel syndrome (29) & healthy controls (26) | Constantinou et al. (2014) [153] | Modified Affect Labeling Task | Physical symptoms (Symptom Checklist), habitual symptom reporting (CSDL), anxiety and depression (HADS) | E | ++ | Viewing negative pictures increased the arousal and gastrointestinal symptoms of both groups. Labeling the pictures did not reduce these effects, although a trend towards less arousal symptoms on the part of patients was observed. A possible confounding variable was reported: the mild affective stimulation used in the experiment. |
| **Beliefs about and attitude to emotions** | | | | | | |
| Chronic fatigue syndrome (121) (21patients measured in pre-post-treatment) & healthy controls (73) | Rimes & Chalder (2010) [133] | Beliefs about Emotions Scale (BES) | Fatigue-related symptoms (CFS), anxiety and depression (HADS), self-sacrifice (YSQ), maladaptive attitudes (DAS-24), perfectionism (PSBS) | CC | + | Patients had significantly more beliefs about the unacceptability of experiencing and expressing emotions than did controls. The scores on the BES had significant positive correlations with measures of negative perfectionism, self-sacrifice, dysfunctional attitudes, depression, anxiety, and fatigue. After psychotherapy a significant reduction of unhelpful beliefs was achieved. |
| Chronic fatigue syndrome (67), close relatives of the patient (44), healthy controls (73) | Brooks, Chalder & Rimes (2017) [132] | Beliefs about Emotions Scale (BES) | Fatigue (CFQ), anxiety and depression (HADS), self-sacrifice (YSQ), perfectionism (PSBS), Goals (RGQ) | CC & retrospective | ++ | Patients’ negative beliefs about emotions were more pronounced than those of healthy controls, especially prior to onset of the syndrome. They retrospectively reported more negative beliefs about emotions in 6 months pre-CFS onset than at the current time. Patients´ and their close relatives’ perceptions of patients’ negative beliefs about emotions were consistent with each other. |
| Chronic fatigue syndrome (80) and healthy controls (80) | Rimes, Ashcroft, Bryan, & Chalder, (2016) [81] | Beliefs about Emotions Scale (BES) | Anxiety and depression (HADS) | E | ++ | Increased negative beliefs about emotions was correlated with greater self-reported suppression. |
| Irritable bowel syndrome (52) | Sibelli, Chalder, Everitt, Workman, et al., (2017) [104] | Semi-structrured interviews |  | CS, qualitative | n.a. (qualitative) | How emotions are experienced was related to beliefs of high expectations of self and social desirability. Beliefs about non-acceptability of expressing negative emotions was highly emphasized, especially by female participants. |
| Medically unexplained symptoms (MUS) (138), MUS comorbid with major depressive disorder (MDD) (114), MDD (106), healthy controls (100) | Schwarz et al. (2017) [116] | Emotion Regulation Skills Questionnaire, (Clarity, Acceptance, Tolerance Subscales) | Physical complaints (SOMS-7T), depression (BDI-II), symptom checklist (SCL-90) | CC | ++ | MUS patients were better than MDD and MUS+MDD patients in: (a) being able to clearly distinguish their emotions, but worse than healthy controls (clarity), (b) accepting their emotions. MUS Patients scored higher in showing tolerance and resilience against strong negative emotions compared to MDD+MUS patients, and lower than healthy controls. |
| Functional dyspepsia (43) & healthy controls (43) | Mazaheri et al. (2016) [136] | Cognitive Emotion Regulation Questionnaire (Acceptance Subscale) | Rome III Interview | CC | ++ | Patients reported less frequent use of acceptance compared to healthy controls. |
| Functional gastrointestinal disorders (167) | Mazaheri (2015) [85] | Difficulties in Emotion Regulation Scale (Clarity, Strategies, and Accept Subscales) | Depression, anxiety and stress (DASS), gastrointestinal symptoms (GSRS) | CS | +++ | Difficulty in access to strategies for dealing with emotions predicted anxiety and stress, while non-acceptance of emotions predicted depression. Lack of emotional clarity and limited access to strategies were correlated with depression, anxiety, and stress, and negatively predicted mindful attention. All the facets of emotion dysregulation were negatively related to mindful attention. |
| Conversion disorders (43) & healthy controls 42) | Del Rio-Casanova et al. (2018) [84] | Difficulties in Emotion Regulation Scale (Clarity and Accept Subscales) | Depression, anxiety (HADS), somatoform dissociation (SDQ-20), psychoform dissociation (DES-II) | CC | +++ | The patient groups had higher scores in lack of emotional clarity and non-acceptance of emotions compared to healthy controls. |
| Psychogenic non- epileptic seizures (70) | Uliaszek et al. (2012) [83] | Difficulties in Emotion Regulation Scale (Clarity, Strategies, and Accept Subscales) | Depression (BDI-II), dissociative experiences (DES), psychological distress (DASS), functioning and physical distress (PHQ-15, DFI), quality of life ( QOLIE-31) | CS | ++ | Two clusters of patients were identified, which differentiated low vs. severe emotion dysregulation. The highly emotion-dysregulated group reported greater difficulties in emotional clarity, acceptance of emotions, and access to emotion regulation strategies, compared to normative data and the low emotion-dysregulated cluster. This group was significantly associated with more psychopathology symptoms as well as higher rates of comorbid psychiatric diagnoses and impairment in quality of life. |
| Psychogenic non- epileptic seizures (43) & epilepsy patients (24) | Brown et al. (2013) [25] | Difficulties in Emotion Regulation Scale (Clarity, Strategies, and Accept Subscales) | Anxiety (GAD-7), depression (PHQ-9), somatization (SDQ- 20), attachment styles (RSQ) | CS | +++ | Two clusters of patients were identified, one of which had more difficulties in emotional clarity, acceptance of emotional responses, access to emotion regulation strategies than the other cluster and the epilepsy patients. They also presented greater alexthymia, psychopathology, and somatization scores than epilepsy patients |
| Psychogenic non- epileptic seizures (56) & healthy controls (88) | Urbanek, et al. (2014) [99] | Beliefs About Emotions Questionnaire | Depression (HADS), seizure characteristics | CC | ++ | Patients evaluated emotions as more overwhelming and uncontrollable, shameful, irrational, contagious, useless, and damaging than did controls. Patients showed more negative beliefs about emotions. Positive correlation was found between negative beliefs about emotions and seizure severity. |
| Psychogenic non- epileptic seizures (71) | Baslet et al. (2017) [135] | 1. Affective Style Questionnaire (adjust & tolerate Subscales)  2. Acceptance and Action Questionnaire | Depression (BDI-II), dissociative experiences (DES), somatic symptoms (PHQ-15) | CS | + | The ability to accept experienced emotions was reduced in the altered responsiveness group (those who do not respond during the seizure) compared to the intact responsiveness patient group. Capacity to tolerate intense emotions was also reduced in the former group. |
| Study 1: rheumatoid arthritis (RA) or osteoarthritis (175) Study 2: fibromyalgia (FA) (89) | Zautra, Smith, Affleck, & Tennen (2001) [154] | Trait Meta-Mood Scale | Daily pain (electronic diary) | CS | + | Mood clarity weakened the inverse relationship between positive and negative affect only in the arthritis group. Mood clarity was related to lower negative affect only in the fibromyalgia group. |
| Fibromyalgia (141) | Veehof et al. (2011) [86] | 1. Five Facet Mindfulness Questionnaire (Non-judge & Describe Subscales) 2. Acceptance and Action Questionnaire-II | Neuroticism and openness to new experiences (NEO- FFI), anxiety and depression (HADS), mental and physical health (SF-12) | CS | + | Being able not to judge experienced feelings and the capacity to describe the feelings was negatively correlated with alexithymia, depression, anxiety, and neuroticism. The facets were not related to physical health. |
| **Reappraisal, automatic thoughts, efficacy in emotion regulation** | | | | | | |
| Chronic pain (128) | Agar-Wilson & Jackson (2012) [134] | Assessing Emotions Scale (Efficacy, Appraisal and Utilization Subscales) | Quality of life (WHOQOL–BRIEF), pain-related disability (ODQ), pain coping (CSQ) | CS | ++ | Efficacy in emotion regulation was associated with higher quality of life. Other dimensions of AES did not contribute to the prediction of pain-related disability. |
| Chronic pain (224) | Wong & Fielding (2013) [101] | Emotion Regulation Questionnaire | Pain (CPQ), pain catastrophizing (PCS) | CS | ++ | Reappraisal ability was negatively associated with pain catastrophizing only in univariate analyses, but not in multivariate analyses, when negative affect and expressive suppression were taken into account. |
| Medically unexplained pain (100) | Chavooshi et al. (2016) [87] | Emotion Regulation Questionnaire | Pain intensity (NPRS), depression & anxiety (DASS- 21), quality of life (QOLI) | I | ++ | Patients treated with intensive short-term dynamic psychotherapy reported increased cognitive reappraisal capacity compared to patients who received treatment as usual. Their other psychosomatic complaints also improved to a greater extent. |
| Somatic symptom disorders (35) & healthy controls (35) | Erkic, Bailer, Fenske, et al., (2017) [100] | Emotion Regulation Questionnaire | Number and intensity of symptoms (SOMS), symptom severity (PHQ-15), life disruption (PDI), depression (BDI) | E | +++ | Patients reported less cognitive reappraisal compared to healthy controls. |
| Medically unexplained symptoms (MUS) (138), MUS comorbid with major depressive disorder (MDD) (114), MDD (106), healthy controls (100) | Schwarz et al. (2017) [116] | Emotion Regulation Skills Questionnaire, (Modification and Self-support Subscales) | Physical complaints (SOMS-7T), depression (BDI-II), symptom checklist (SCL-90) | CC | ++ | MUS patients scored higher in their efficacy beliefs about modifying negative emotions compared to MUS+MDD patients and scored lower than healthy controls. MUS patients scored higher in being able to support oneself in emotionally demanding situations compared to MDD or MUS+MDD patients and lower than controls. |
| Fibromyalgia (403) & healthy controls (196) | van Midden­dorp et al. (2008) [96] | Emotion Regulation Questionnaire | Pain (MPI), fibromyalgia impact (FIQ), mental distress (MPI & FIQ) | CC | ++ | Reappraisal ability did not differ between groups. |
| Fibromyalgia (403) | Geenen et al. (2012) [102] | Emotion Regulation Questionnaire | Fibromyalgia impact (FIQ) | CS | ++ | Cognitive reappraisal was not related to fibromyalgia impact. |
| Psychogenic non- epileptic seizures (72) & healthy controls (72) | Gul & Ahmad (2014)[78] | Emotion Regulation Questionnaire | Psychological distress (DASS) | E | +++ | The patient group demonstrated reduced cognitive reappraisal, which was also associated with reduced cognitive flexibility. |
| Functional neurological symptoms (19) & healthy controls (19) | Kienle et al. (2018) [137] | Emotion Regulation Questionnaire | Functional neurological symptoms (SDQ-20), psychological strain (SCL 90R) | E | ++ | Patients reported less of a tendency to use cognitive reappraisal than controls. |
| Tension-type headache (105) & healthy controls | Yucel et al. (2002) [138] | Automatic Thoughts Scale | Depression (BDI), assertiveness (RAS) | CC | +++ | Patients had more frequent automatic negative thoughts, which were associated with higher depression scores. Chronic tension-type headache patients had more depression and negative automatic thoughts than episodic tension-type headache patients. |
| Irritable bowel syndrome (14) & healthy controls (14) | Kilkens, Honig, van Nieuwen­­hoven, Riedel, & Brummer (2004) [155] | Affective Memory Performance Test | Experimental reduction of serotonin, visceral sensitivity, psychological functioning (HAMD, SCL-90, HADS) | E | +++ | The reduction in serotonin synthesis caused impaired recall of positive words, but not of negative and neutral words in both patients and controls. The reduction of serotonin synthesis also increased pain and the urge to defecate and lowered the perceptual threshold. |
| Functional dyspepsia (43) & healthy controls (43) | Mazaheri et al. (2016) [136] | Cognitive Emotion Regulation Questionnaire, Positive reappraisal, Rumination and Other-blame Subscales | Rome III Interview | CC | ++ | Patients reported less frequent use of positive reappraisal, but more use of rumination and other-blame compared to healthy controls. |

^1^Quality of the studies was rated with +, ++, or +++ when 25–49%, 50–79%, or 80% or more of the criteria were rated with “yes.”

^2^ The two article findings are based on the same sample data.

**Abbreviations of the study designs**

**CS:** Cross Sectional   **CC:** Case Control   **E:** Experimental   **L:** Longitudinal   **I:** Intervention/psychotherapy study

**Abbreviations of the symptom measures & paradigms**

**BAI:** Beck Anxiety Inventory, **BDI:** Beck Depression Inventory, **BSI:** The Brief Symptom Inventory, **CAS:** Colored Analog Scale, **CES-D:** Center for Epidemiology Studies-Depression Scale, CFQ: The Chalder Fatigue Questionnaire, **CPQ:** Chronic Pain Questionnaire, **CSDL:** Checklist for Symptoms in Daily Life, **CSQ:** The Coping Strategies Questionnaire, **DAS:** The Dyadic Adjustment Scale, **DASS:** Depression, Anxiety and Stress Symptoms, **DES:** Dissociative Experiences Scale, **DFI:** Disruption of Functioning Index, **FABQ:** Fear-Avoidance Belief Questionnaire, **FIQ:** The Fibromyalgia Impact Questionnaire, **FBL:** Freiburger Beschwerdeliste-Revised, **GSRS:** Gastrointestinal Symptom Rating Scale, **GAD:** Generalized Anxiety Disorder Questionnaire, **HADS:** Hospital Anxiety and Depression Scale, **HAM-A & -D:** Hamilton Rating Scale for Depression & Anxiety, **MADRS:** Montgomery and Asberg Depression Rating Scale, **MASC:** Movie for the Assessment of Social Cognition, **MINI:** Mini International Neuropsychiatric Interview, **MPI:** Multidimensional Pain Inventory, **MPQ:** McGill Pain Questionnaire, **MSS:** Mental States Stories Test , **NEO-FFI:** NEO Five-Factor Personality Inventory, **NPRS:** Numerical Pain Rating Scale, **ODQ:** Oswestry Disability Questionnaire, **PANAS:** Positive and Negative Affect Scale, **PCS:** The Pain Catastrophizing Scale, **PHQ:** The Patient Health Questionnaire, **PSBS:** Perfectionistic Self-belief Scales, **RAS:** Rathus Assertiveness Schedule, RGQ: The Roles and Goals Questionnaire, **RSQ:** Responses to Stress Questionnaire, **QOLI:** Quality of Life Inventory, **SAS:** Somatosensory Amplification Scale, **SCID:** Structured Clinical Interview for Personality Disorders, **SCL-90:** Symptom Checklist-90, **SDQ:** Somatoform Dissociation Questionnaire, **SF:** Short form Health Survey, **SOMS:** Screening for Somatoform Disorders, **STAI:** State Trait Anxiety Inventory, **WHOQOL–BRE:** World Health Organization Quality of Life Scale-brief, **YSQ:** Young Schema Questionnaire
